# Identification of core genes as potential biomarkers for predicting progression and prognosis in glioblastoma

**DOI:** 10.3389/fgene.2022.928407

**Published:** 2022-09-27

**Authors:** Jianping Zeng, Shushan Hua, Jing Liu, Rajneesh Mungur, Yongsheng He, Jiugeng Feng

**Affiliations:** ^1^ Department of Neurosurgery, The First Affiliated Hospital of Nanchang University, Nanchang, China; ^2^ Department of Neurobiology, Zhejiang University School of Medicine, Hangzhou, China; ^3^ Department of Pharmacy, The First Affiliated Hospital of Nanchang University, Nanchang, China; ^4^ Department of Neurosurgery, The First Affiliated Hospital of Zhejiang University, Hangzhou, China

**Keywords:** glioblastoma, expression profiling data, hub genes, prognosis, NDC80

## Abstract

**Background:** Glioblastoma is a common malignant neuroepithelial neoplasm with poor clinical outcomes and limited treatment options. It is extremely important to search and confirm diverse hub genes that are effective in the advance and prediction of glioblastoma.

**Methods:** We analyzed GSE50161, GSE4290, and GSE68848, the three microarray datasets retrieved from the GEO database. GO function and KEGG pathway enrichment analyses for differentially expressed genes (DEGs) were performed using DAVID. The PPI network of the DEGs was analyzed using the Search Tool for the Retrieval of Interacting Genes database and visualized by Cytoscape software. Hub genes were identified through the PPI network and a robust rank aggregation method. The Cancer Genome Atlas (TCGA) and the Oncomine database were used to validate the hub genes. In addition, a survival curve analysis was conducted to verify the correlation between the expression of hub genes and patient prognosis. Human glioblastoma cells and normal cells were collected, and then RT-PCR, Western blot, and immunofluorescence were conducted to validate the expression of the *NDC80* gene. A cell proliferation assay was used to detect the proliferation of glioma cells. The effects of *NDC80* expression on migration and invasion of GBM cell lines were evaluated by conducting scratch and transwell assays.

**Results:** A total of 716 DEGs were common to all three microarray datasets, which included 188 upregulated DEGs and 528 downregulated DEGs. Furthermore, we found that among the common DEGs, 10 hub genes showed a high degree of connectivity. The expression of the 10 hub genes in TCGA and the Oncomine database was significantly overexpressed in glioblastoma compared with normal genes. Additionally, the survival analysis showed that the patients with low expression of six genes (*BIR5C*, *CDC20*, *NDC80*, *CDK1*, *TOP2A*, and *MELK*) had a significantly favorable prognosis (*p* < 0.01). We discovered that *NDC80*, which has been shown to be important in other cancers, also has an important role in malignant gliomas. The RT-PCR, Western blot, and immunofluorescence results showed that the expression level of NDC80 was significantly higher in human glioblastoma cells than in normal cells. Moreover, we identified that NDC80 increased the proliferation and invasion abilities of human glioblastoma cells.

**Conclusion:** The six genes identified here may be utilized to form a panel of disease progression and predictive biomarkers of glioblastoma for clinical purposes. *NDC80*, one of the six genes, was discovered to have a potentially important role in GBM, a finding that needs to be further studied.

## Introduction

Glioblastoma multiforme (GBM) is considered the most malignant brain tumor, with high proliferative capacity and invasion characteristics that result in rapid progression and a high degree of malignancy. GBM is classified as grade IV by the World Health Organization (WHO), and the mortality rate of patients in the first year after diagnosis is close to 80% (Strepkos et al., 2020). GBM is also the most common and fatal primary malignant brain tumor in adults (Bi et al., 2020), and the 5-year survival rate of patients diagnosed with GBM is less than 6% (Sabbagh et al., 2020). Currently, the standard treatment for GBM is surgical resection followed by radiotherapy with or without concurrent adjuvant temozolomide chemotherapy (Stupp et al., 2002; Tan et al., 2020). Tumor-treating fields, delivering low-intensity alternating electric fields, can also be used concurrently with adjuvant temozolomide. While there have been many reports on immunotherapy and gene therapy for GBM, the effects are not completely confirmed due to the inconsistency in treatment methods and evaluation criteria. Gene expression profiling that provides rich data on genetics, gene expression, and promoter methylation can aid in the early diagnosis and validation of specific biomarkers (Cloughesy et al., 2014). However, several studies using a single or small sample dataset for gene expression analysis require further reproducibility testing and independent validation or experimental studies (De Preter et al., 2010; He et al., 2015; Cheng et al., 2016). In order to generate robust results, we performed gene expression profiling analysis across different expression datasets to explore sensitive and specific biological markers for early diagnosis and validation of interventions among GBM patients. Furthermore, we conducted experiments using quantitative real-time PCR, Western blotting analysis, and immunofluorescence between GBM and normal control cells, with the purpose of verifying a newly identified biological marker.

## Materials and methods

**FIGURE 1:**
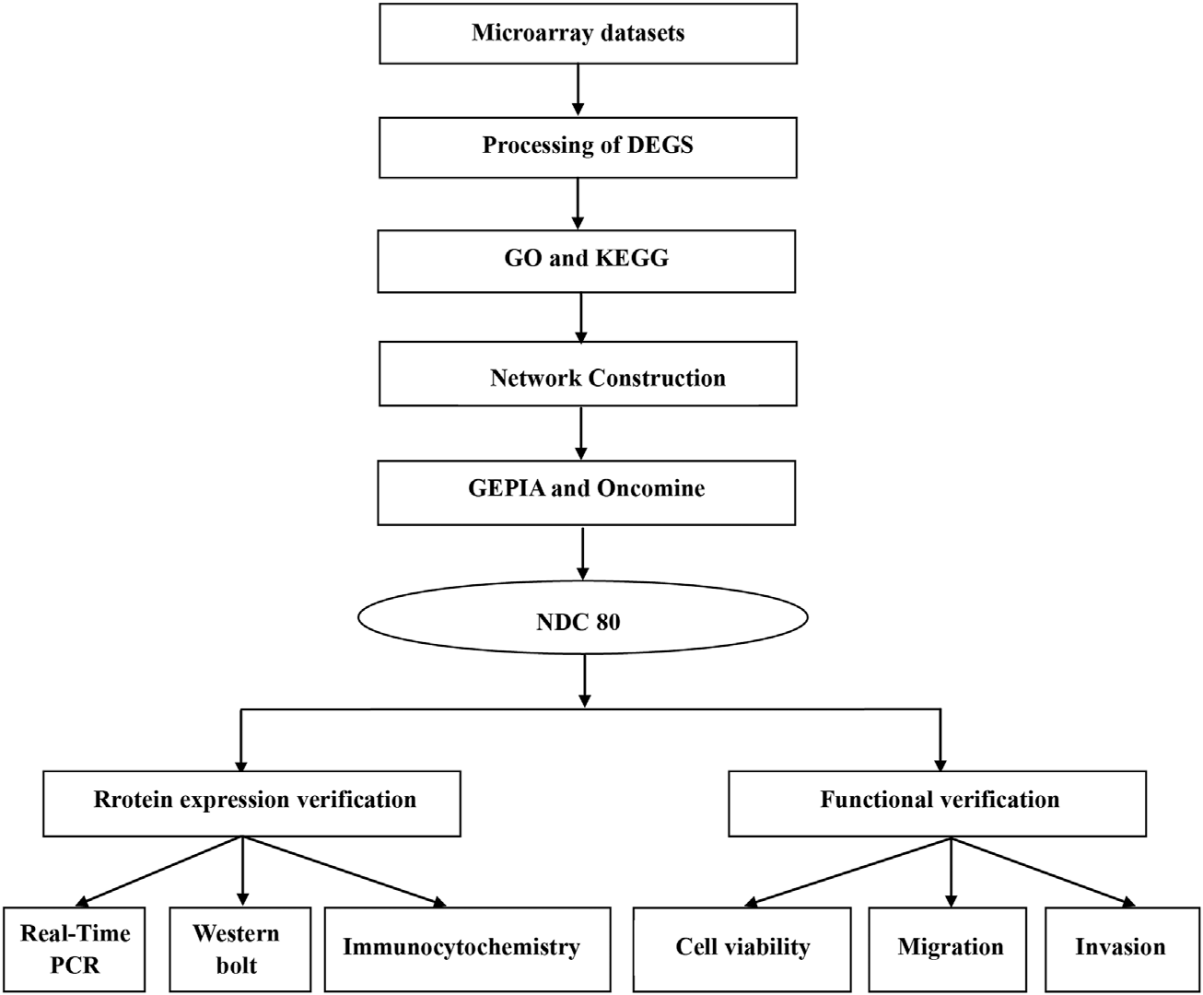
Workflow chart to better represent the analyses performed and to link some of the methods across the fields in this study.

First, we provide a workflow chart related to this study to make the research idea of this study clearer (Figure 1).

### Microarray data source

Gene expression datasets were obtained from the Gene Expression Omnibus (GEO) database (https://www.ncbi.nlm.nih. gov/geo/). There were 50,044 series on human GBM cancer retrieved from the GEO database. After a careful review, three gene expression profiles (GSE50161, GSE4290, and GSE68848) were selected. All of them are based on platform GPL570 [(HG-U133_Plus_2) Affymetrix Human Genome U133 Plus 2.0 Array]. All the data were freely available online.

### Data processing of DEGs

The DEGs between GBM and normal samples were identified by using the online analysis tool GEO2R (https://www.ncbi.nlm. nih.gov/geo/geo2r/) for the GEO database, based on the R language. We considered DEGs as differentially expressed if genes met the cutoff criteria of an adjusted *p* < 0.05 and |logFC| ≥ 2.0 (Ward et al., 2013; Saura et al., 2014). The Venn diagram webtool (bioinformatics.psb.ugent.be/webtools/Venn/) was used to identify the overlapping DEGs between datasets.

### GO and KEGG pathway analysis of DEGs

GO functional and KEGG pathway enrichment analyses were performed using the Database for Annotation, Visualization, and Integrated Discovery (DAVID v6.8, http://david-d.ncifcrf.gov/) to identify the DEG pathways (Huang et al., 2009). *p* < 0.05 was considered statistically significant.

### Protein–protein interaction network construction and hub gene identification

The STRING database (v11.5, http://string-db.org/) aims to collect and integrate the knowledge of all functional interactions between expressed proteins by consolidating known and predicted protein–protein association data for a large number of organisms (Szklarczyk et al., 2017). We uploaded the overlapping DEGs to the online STRING database and obtained the data for the PPI network. Next, we used Cytoscape software (v3.9.0) to construct a PPI network. We screened the PPI network modules using the MCODE plug-in in Cytoscape. The default settings were used, with a degree cutoff set to 2, node core cutoff set to 0.2, K-core set to 2, and max depth set to 100. Generally, we always denote the most highly connected genes in the PPI network as hub genes, which are expected to play an important role in understanding the biological mechanism of a response under specific conditions (Das et al., 2017). In order to identify the hub genes in this study, CytoHubba, a Cytoscape plug-in, was used to analyze the data obtained from STRING. Finally, we obtained the top 10 hub genes.

### Validation of hub gene expression levels

Gene Expression Profiling Interactive Analysis (GEPIA, v2017, http://gepia.cancer-pku.cn) and Oncomine (https://www.oncomine.org) were used to validate the expression of the candidate hub genes (Giordano et al., 2009; Tang et al., 2017).

### Survival analysis of hub genes

The gene expression data of 325 patients (203 males and 122 females), with an average age of 43.38 years, were downloaded from the CGGA (http://www.cgga.org.cn). Patients were categorized into either a high-expression group or a low-expression group according to the expression level of the 10 hub genes. We regarded OS as the prognostic outcome of patients with glioblastoma.

### Cell culture

Human glioblastoma U251 and U-87MG cells and normal control HA1800 cells were provided by G. F. Vande Woude, Van Andel Research Institute, Grand Rapids, MI. The cells were cultured in Dulbecco’s modified Eagle medium containing 10% fetal bovine serum and placed in a 5% carbon dioxide and 37°C cell culture incubator.

### Quantitative real-time PCR

Total RNA was extracted from cells by using TRIzol reagent (Invitrogen, United States) following the manufacturer’s protocol. A cDNA synthesis kit (Takara, China) was used for the synthesis of cDNA according to the manufacturer’s instructions. RT-PCR was performed using a PrimeScript II reverse transcription kit from Takara. The primer sequence was as follows: NDC80 sense strand: 5′-ATC​AAG​GAC​CCG​AGA​CCA​CT-3′, NDC80 antisense strand: 5′-ATG​TAT​GAG​GAG​CCC​CCA​CT-3′; β-actin sense strand: 5′-CTG​GAA​CGG​TGA​AGG​TGA​CA-3′, and β-actin antisense strand: 5′-CGG​CCA​CAT​TGT​GAA​CTT​TG -3′.

### Western blot assay

Cells were lysed, and the protein in the supernatant extracts was quantified using a BCA protein assay kit (Beyotime Institute of Biotechnology). Fifty micrograms per lane of total cell lysates were resolved on sodium dodecyl sulfate-polyacrylamide gel electrophoresis gels and transferred onto polyvinylidene fluoride membranes (Millipore, Billerica, MA, United States). Membranes were incubated with the primary antibody overnight at 4°C. The next day, the membranes were incubated with a horseradish peroxidase–linked secondary anti-rabbit or anti-mouse antibody (Bio-Rad). Immunoreactivity was detected using enhanced chemiluminescence (Amersham Biosciences, Piscataway, NJ, United States) with a Chemidoc imaging system and Quantity One software (Bio-Rad). A densitometric analysis was performed by using the Quantity One software. β-actin was used as a loading control. NDC80 antibodies were purchased from Abcam, United States.

### Immunofluorescence

Cells were seeded on coverslips and incubated for 24 h under normoxic conditions. Subsequently, the cells were fixed with 4% paraformaldehyde at room temperature and permeabilized with 0.2% Triton X-100 for 10 min. Next, cells were washed with phosphate-buffered saline (PBS) and blocked in PBS containing 5% bovine serum albumin (Sigma-Aldrich, St. Louis, MO, United States) for 90 min. The cells were washed with PBS, and the primary antibody rabbit anti-NDC80 (1:200; Abcam, United States) was diluted with PBS containing 2% bovine serum albumin and incubated overnight at 4°C. The cells were washed with PBS and incubated for 2 h with an anti-rabbit fluorescent secondary antibody (Amersham) at room temperature. Finally, DAPI (Beijing ComWin Biotech Co., Ltd., China) was added to each sample for nuclear counterstaining. The cells were observed and photographed using an Olympus BX61WI-FV1200MPE confocal microscope to show representative cells.

### Cell proliferation assay

Cell viability was measured using a Cell Counting Kit-8 (CCK-8) assay (Dojindo Molecular Laboratories, Inc.) according to the manufacturer’s instructions. Transfection with NDC80 or the non-specific control was performed in 96-well plates in quadruplicate. The cell culture medium was replaced at 24 h following transfection. CCK-8 (10 µl) was added to each well, and the absorbance at 450 nm was measured following incubation for 2 h at 37°C. Each experiment was repeated in triplicate.

### Scratch assay

Briefly, cells were grown to full confluence in 6-well culture plates. After reaching confluency, monolayers were scratched with a sterile pipette tip to make a scratch of approximately 0.4–0.5 mm in width, and cells were cultured in a serum-deprived medium. After 24 h, the wound gap was observed, and images were captured. All scratch assays were performed in triplicate.

### Transwell invasion assay

Transwell invasion assays were performed with 24-well Matrigel-coated chambers (8-μm pore size) from BD Biosciences. According to the manufacturer’s protocol, cells were permitted to grow to 75–80% confluence and then were serum starved for 24 h. Next, the nonmotile cells were removed with a cotton swab. The remaining cells at the lower surface of the filter were fixed with cold methanol and stained with 0.1% (w/v) crystal violet (Sigma). The invading cells were quantified by counting 10 random fields at × 200 magnification. All transwell invasion assays were performed in three independent experiments.

### Statistical analysis

In this study, each experiment was carried out at least three separate times. Data are presented as mean ± standard error of mean (SEM). Statistical tests were performed using SPSS version 19.0 software for Windows (SPSS Inc., Chicago, United States). Two-tailed Student’s *t*-test was used for comparisons between groups. *p* < 0.05 was considered to be a significant difference.

## Results

### Identification of differentially expressed genes

**FIGURE 2:**
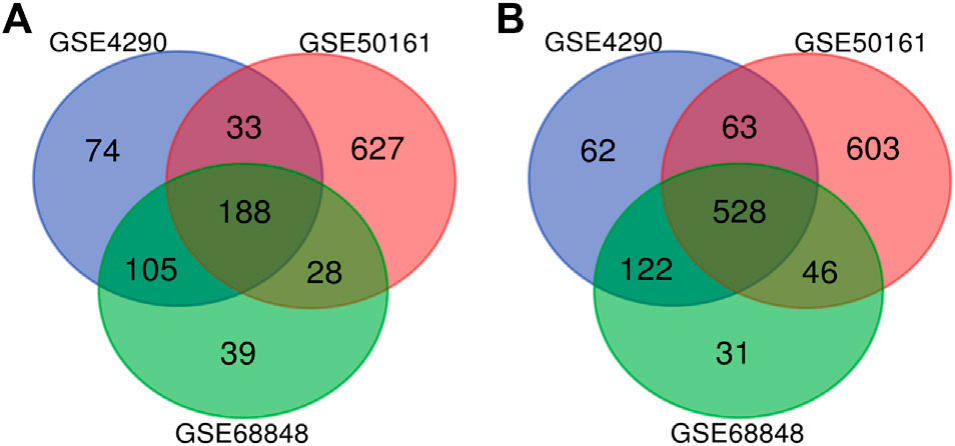
Use the Venn diagram to obtain DEGs common to all three GEO datasets. The red, blue, and green colors represent GSE4290, GSE50161, and GSE68848, respectively; 716 DEGs were identified as significantly differentially expressed among all three groups. **(A)** 188 upregulated common genes were found in the three datasets. **(B)** 528 downregulated genes were found in the three datasets. DEGs: differentially expressed genes.

Our study consisted of three datasets: GSE50161, GSE4290, and GSE68848. GSE50161 contained 34 GBM and 13 normal samples, GSE4290 contained 77 GBM and 23 normal samples, and GSE68848 contained 228 GBM and 28 normal samples (Table 1). Based on the cutoff criteria of *p* < 0.05 and |logFC| ≥ 2, a total of 2,116 DEGs were identified from GSE50161, including 876 upregulated genes and 1,240 downregulated genes. There were 1,175 DEGs identified in gene chip GSE4290; 400 genes were upregulated, and 775 genes were downregulated. GSE68848 had 1,087 DEGs, including 360 upregulated genes and 727 downregulated genes. A Venn analysis was performed to find the overlap of the DEGs (Figure 2). Finally, 716 DEGs were identified as significantly differentially expressed among all three groups, of which 188 were significantly upregulated and 528 were downregulated genes.

**TABLE 1:** Statistics of the three microarray databases.

Dataset ID	GBM	Normal	Total number
GSE50161	34	13	47
GSE4290	77	23	100
GSE68848	228	28	256

### Gene Ontology enrichment analysis and Kyoto Encyclopedia of Genes and Genomes pathway analysis of DEGs

We performed GO analysis of the 716 DEGs mutual to all three groups and determined that the genes were mainly associated with biological information transfer or cellular function alteration, including chemical synaptic transmission, neurotransmitter secretion, nervous system development, regulation of exocytosis, glutamate secretion, cell junction, synaptic vesicle membrane, axon, synapse, synaptic vesicle, calcium ion binding, and calcium-dependent phospholipid binding.

Furthermore, the results of KEGG analysis showed that the mutual DEGs were mainly enriched in retrograde endocannabinoid signaling, GABAergic synapse, morphine addiction, calcium signaling pathway, and synaptic vesicle cycle (Table 2).

**TABLE 2:** Top 5 significantly enriched GO terms and KEGG pathways of DEGs.

Category	Term	Gene function	Count	p-value
GO enrichment analysis				
GOTERM_BP_DIRECT	GO:0007268	Chemical synaptic transmission	52	1.43E-22
GOTERM_BP_DIRECT	GO:0007269	Neurotransmitter secretion	16	2.24E-07
GOTERM_BP_DIRECT	GO:0007399	Nervous system development	36	2.48E-07
GOTERM_BP_DIRECT	GO:0017157	Regulation of exocytosis	12	7.72E-07
GOTERM_BP_DIRECT	GO:0014047	Glutamate secretion	11	3.46E-05
GOTERM_CC_DIRECT	GO:0030054	Cell junction	81	3.26E-30
GOTERM_CC_DIRECT	GO:0030672	Synaptic vesicle membrane	22	1.55E-13
GOTERM_CC_DIRECT	GO:0030424	Axon	39	7.77E-13
GOTERM_CC_DIRECT	GO:0045202	Synapse	34	9.31E-12
GOTERM_CC_DIRECT	GO:0008021	Synaptic vesicle	24	1.10E-10
GOTERM_MF_DIRECT	GO:0005509	Calcium ion binding	60	1.05E-06
GOTERM_MF_DIRECT	GO:0005544	Calcium-dependent phospholipid binding	13	8.79E-04
KEGG pathway analysis				
KEGG_PATHWAY	hsa04723	Retrograde endocannabinoid signaling	25	5.47E-11
KEGG_PATHWAY	hsa04727	GABAergic synapse	22	1.00E-09
KEGG_PATHWAY	hsa05032	Morphine addiction	22	4.23E-09
KEGG_PATHWAY	hsa04020	Calcium signaling pathway	29	2.31E-08
KEGG_PATHWAY	hsa04721	Synaptic vesicle cycle	17	3.78E-07

### PPI network construction and hub gene identification

**FIGURE 3:**
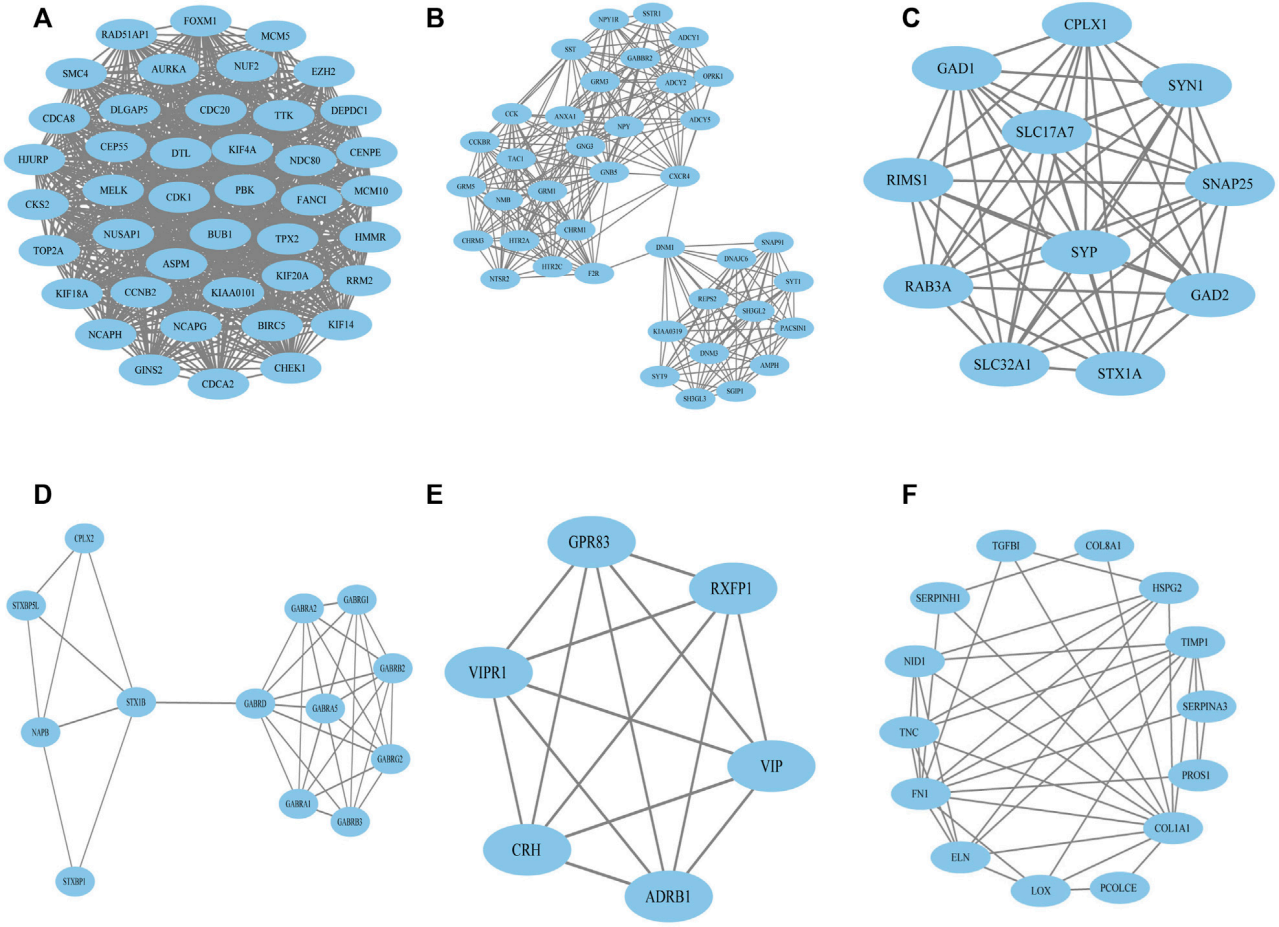
Top six primary modules of PPI sub-networks from the MCODE in Cytoscape software. **(A)** Module 1; **(B)** module 2; **(C)** module 3; **(D)** module 4; **(E)** module 5; **(F)** module 6. PPI: protein–protein interaction; MCODE: Molecular Complex Detection.

**FIGURE 4:**
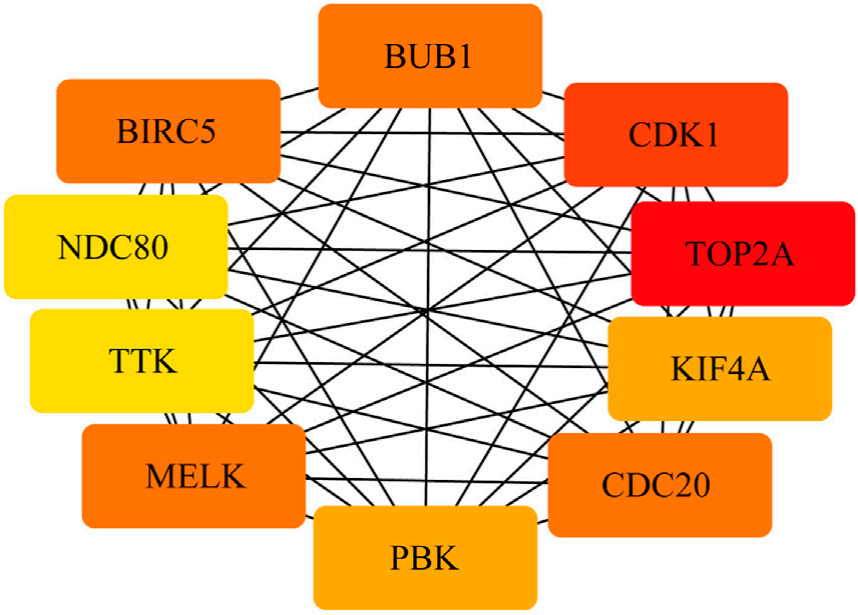
PPI network of the top 10 hub genes. There are close interrelationships among the 10 hub genes.

To construct the network of the PPI relationships of the DEGs, we used the online tool STRING. We only retained the PPI relationships that had a combined score > 0.4. Based on the information obtained from the STRING database, we produced a network diagram. There were 501 nodes and 3,030 edges in the network. Cytoscape was used to evaluate the primary modules of the PPI sub-network using the MCODE plug-in for molecular complex detection (Figure 3). There were 42 genes in subnetwork a, 39 genes in subnetwork b, 11 genes in subnetwork c, 13 genes in subnetwork d, 6 genes in subnetwork e, and 14 genes in subnetwork f. Based on the node degree, we calculated the top 10 hub genes among the 716 DGEs (Table 3) using CytoHubba in Cytoscape. The top 10 genes were also constructed into a network (Figure 4). The gene with the highest degree was DNA topoisomerase II alpha (*TOP2A*; degree = 78), followed by cyclin-dependent kinase 1 (*CDK1*; degree = 64), budding uninhibited by benzimidazole 1 (*BUB1*; degree = 53), cell division cycle protein 20 (*CDC20*; degree = 53), baculoviral IAP repeat-containing 5 (*BIRC5*; degree = 53), maternal embryonic leucine zipper kinase (*MELK*; degree = 53), kinesin family member 4A (*KIF4A*; degree = 52), PDZ binding kinase (PBK; degree = 52), NDC80 kinetochore complex component (*NDC80*; degree = 51), and TTK protein kinase (*TTK*; degree = 51). All of them were upregulated genes.

**TABLE 3:** Top ten hub genes with a higher degree of connectivity.

Rank	Name	Gene description	Score
1	TOP2A	DNA topoisomerase II alpha	78
2	CDK1	Cyclin-dependent kinase 1	64
3	BUB1	Budding uninhibited by benzimidazole 1	53
4	CDC20	Cell division cycle protein 20	53
5	BIRC5	Baculoviral IAP repeat-containing 5	53
6	MELK	Maternal embryonic leucine zipper kinase	53
7	KIF4A	Kinesin family member 4A	52
8	PBK	PDZ binding kinase	52
9	NDC80	NDC80 kinetochore complex component	51
10	TTK	TTK protein kinase	51

### Assessment of ten hub genes in TCGA and Oncomine databases

**FIGURE 5:**
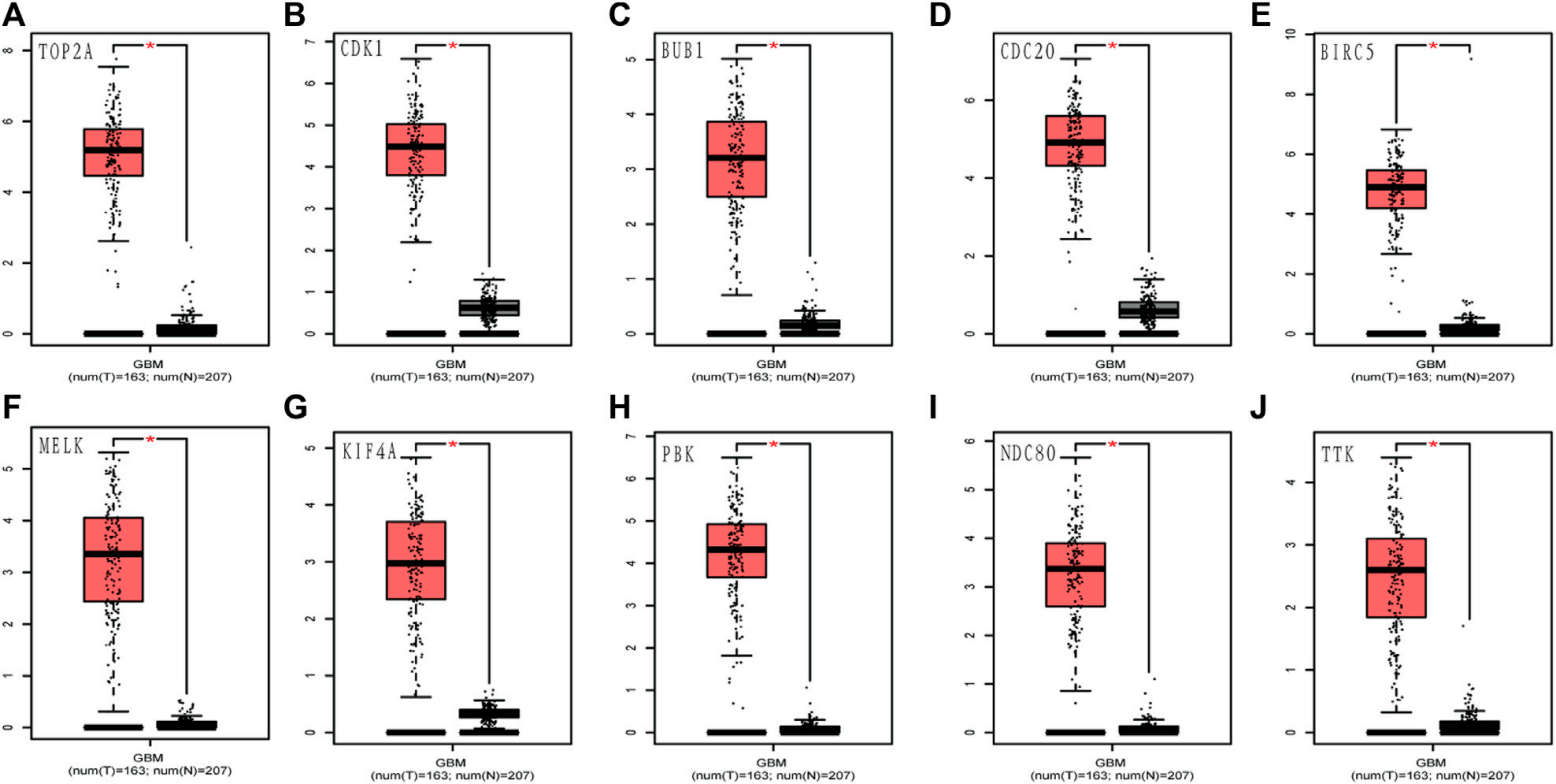
Validation of the 10 hub gene expression levels between normal brain and GBM samples from TCGA and GTEx data in GEPIA. All of the 10 hub genes were found to be upregulated. **(A)** *TOP2A*, **(B)** *CDK1*, **(C)** *BUB1*, **(D)** *CDC20*, **(E)** *BIRC5*, **(F)** *MELK*, **(G)** *KIF4A*, **(H)** *PBK*, **(I)** *NDC80*, and **(J)** *TTK*. TCGA: The Cancer Genome Atlas. *p < 0.05.

**FIGURE 6:**
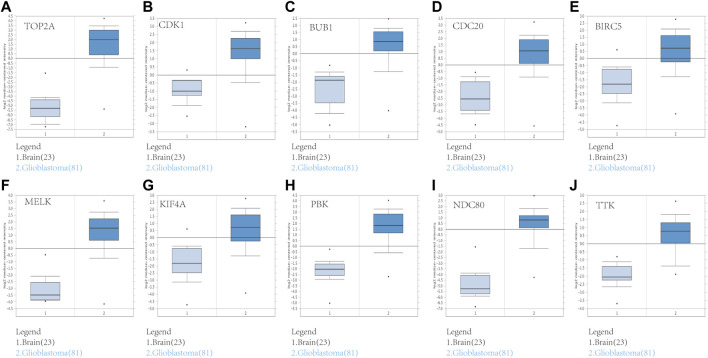
Validation of the 10 hub gene expression levels between normal brain and GBM samples based on Oncomine data. All of the 10 hub genes were confirmed to be upregulated. **(A)** *TOP2A*, **(B)** *CDK1*, **(C)** *BUB1*, **(D)** *CDC20*, **(E)** *BIRC5*, **(F)** *MELK*, **(G)** *KIF4A*, **(H)** *PBK*, **(I)** *NDC80*, and **(J)** *TTK*. *p < 0.05.

To assess the roles of these 10 hub genes in GBM, gene expression validations were performed. All of the 10 hub genes were found to be upregulated in The Cancer Genome Atlas (TCGA) and Oncomine databases (Figures 5, 6).

### Survival analysis of ten hub genes

**FIGURE 7:**
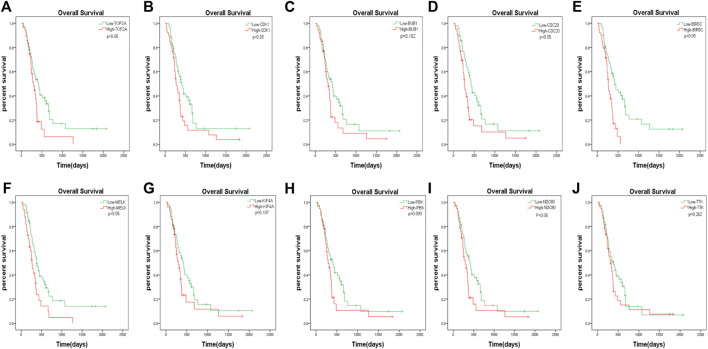
The prognostic values of hub genes in GBM. High mRNA level of any one gene of *TOP2A*, *CDK1*, *CDC20*, *BIRC5*, *MELK*, and *NDC80* was associated with overall survival (OS) in all GBM patients *p* < 0.05. Four hub genes (*BUB1*, *KIF4A*, *PBK* and *TTK*) had no statistical significance between gene expression and the clinical outcome of GBM.

The GBM patients enrolled in the study were each treated by members of the Chinese Glioma Genome Atlas (CGGA) group. Tumor tissue samples were collected at the time of each patient’s surgery after informed consent. Neuropathologists established the diagnosis and ensured the quality of the tissue for molecular testing. Overall survival (OS) was calculated from the date of diagnosis until death or the end of follow-up. The date of death was defined by death certificates, which were obtained from local hospitals and police stations. We assessed the prognostic effect of the 10 hub genes. According to the analysis, we found that only the downregulation of *TOP2A*, *CDK1*, *CDC20*, *BIRC5*, *MELK*, and *NDC80* was closely associated with a decreased OS among patients with GBM (Figure 7). The remaining four hub genes (*BUB1*, *KIF4A*, *PBK*, and *TTK*) had no statistical significance between gene expression and the clinical outcome of GBM (Figure 7).

### *NDC80* expression in human glioblastoma cells is increased compared with normal cells

**FIGURE 8:**
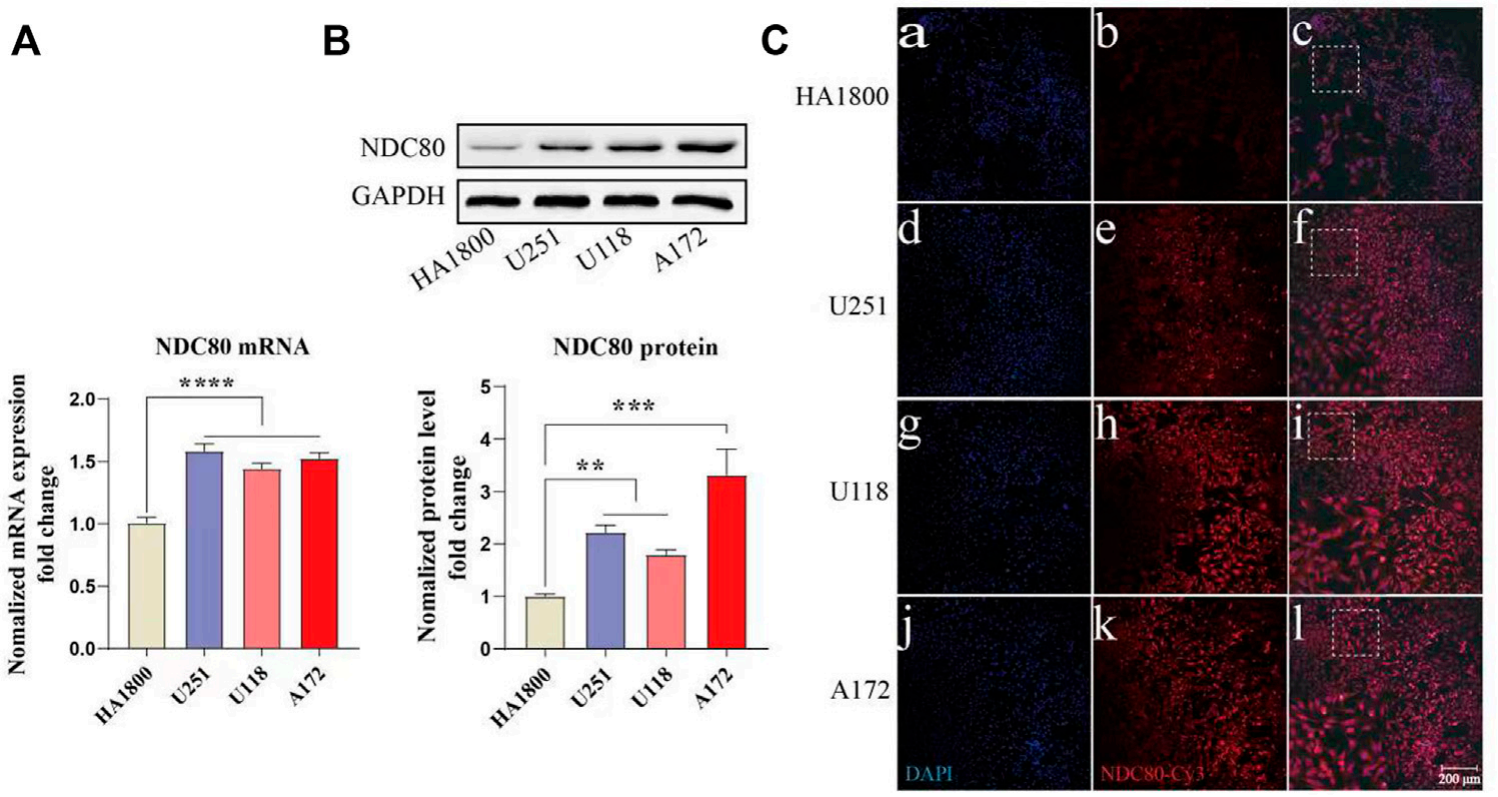
Expression of NDC80 in human glioblastoma cells and normal cells. **(A)** Transcriptional levels of NDC80 in human glioblastoma cells and normal cells. Statistical analysis was performed by using one-way ANOVA with Bonferroni’s multiple comparison test; *F*_3, 32_ = 28.59. *n* = 3 tests on 3 samples per group. **(B)** Translational levels of NDC80 in human glioblastoma cells and normal cells. Statistical analysis was performed by using an unpaired *t*-test *n* = 3 samples per group. **(C)** Immunofluorescence of NDC80. **(b)**, **(e)**, **(h)** and **(k)** Cy3-immuno fluorescence (red) indicates NDC80 was observed in cells. **(a)**, **(d)**, **(g)** and **(j)** DAPI (blue) indicates nuclear staining in cells. **(c)**, **(f)**, **(i)** and **(l)** merged image (magnification × 100). Data are presented as mean ± SEM. **p < 0.01, ***p < 0.001, and ****p < 0.0001.

NDC80 has an important role in other cancers, such as liver and breast cancer, but it is rarely reported in GBM. Our study found that *NDC80* was highly expressed in GBM. Then, we further examined the expression of *NDC80* in different malignant glioma cells. *NDC80* mRNA levels were determined by quantitative real-time reverse transcription-polymerase chain reaction (RT-PCR) between human glioblastoma cells and normal control cells (Figure 8A). RT-PCR showed that *NDC80* was upregulated significantly (*p* < 0.001) in human glioblastoma cells when compared with normal astrocytes (HA 1800). In addition, we found that the translation level of *NDC80* (*p* < 0.001) was also significantly increased in malignant glioma cells, as determined by Western blotting analysis (Figure 8B). In order to further substantiate these findings, we also observed the protein expression of *NDC80* in cells by immunofluorescence. The results of immunofluorescence were consistent with the results of RT-PCR and Western blotting, as NDC80 protein was visualized by immunofluorescence in cells. U251 and U-87MG cells displayed higher red fluorescence than HA1800 cells (Figure 8C), suggesting that *NDC80* expression in malignant glioma cells is higher than in normal cells.

### *NDC80* upregulates the proliferation and invasion abilities of human glioblastoma cells

**FIGURE 9:**
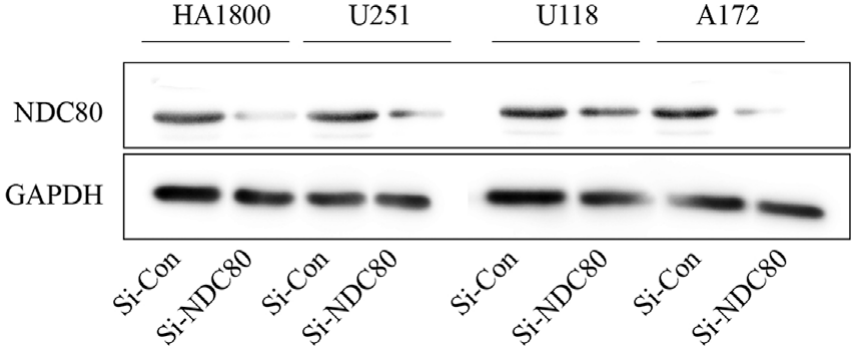
Si-NDC80 can inhibit the protein translation level of human glioblastoma cells and normal astrocytes NDC80. Western blotting detected NDC80 knockdown, NDC80 protein expression levels in human glioblastoma U251, U118, and A172 cell lines, and normal astrocyte cell line HA1800 were significantly downregulated.

**FIGURE 10:**
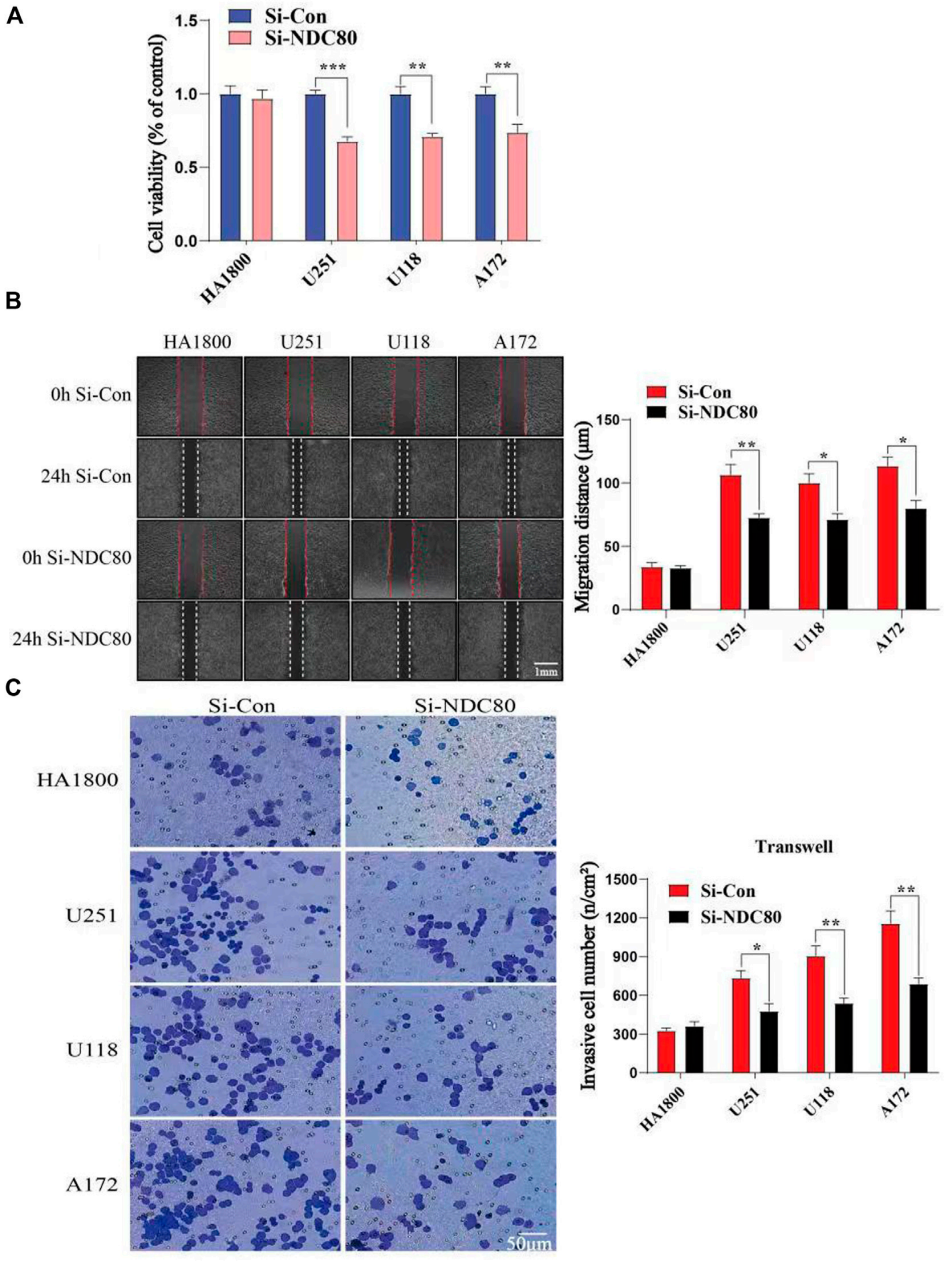
Compare the cell viability (OD value) and invasion ability of human glioblastoma cells with normal astrocytes in each group. **(A)** CCK-8 test suggests that Si-NDC can downregulate the cell viability of human glioblastoma cells; *n* = 5 samples per group; unpaired *t*-test. **(B)** Scratch assay showed that the migration ability of human glioblastoma cells (U251, U118, and A172) in the Si-NDC80 group was significantly lower than that of normal astrocytes; *n* = 4 tests per group; unpaired *t*-test. **(C)** Number of invasive cells in the Si-NDC80 group of transwell invading experimental human glioblastoma cells (U251, U118, and A172) was significantly lower than that of the control; *n* = 5 tests per group; unpaired *t*-test. Data are presented as mean ± SEM. *p < 0.05, **p < 0.01, and ***p< 0.001.

Western blotting experiments confirmed that the siNDC80 designed by us could knock down the expression of NDC80 protein in cells (Figure 9). Additionally, the CCK-8 test results showed that the proliferation ability of U251, U118, and A172 cells after siNDC80 treatment was lower than that of the control cells (*p* < 0.05). However, there was no significant difference between the siNDC80-treated normal astrocytes (HA 1800) and their control (*p* > 0.05), as shown in Figure 10A. Collectively, the results demonstrated that the proliferation ability of GBM cells was significantly affected by the expression of NDC80 protein. In order to determine the role of NDC80 in GBM migration, we first conducted a scratch test and a transwell invasion test. As shown in the scratch test results (Figure 10B), knockdown of NDC80 protein reduced the migration ability of human glioblastoma cells (U251, U118, and A172) when compared with negative control cells. As shown in the results of the transwell invasion experiment in Figure 10C, the number of invasive cells following siNDC80 treatment of human glioblastoma cells (U251, U118, and A172) was significantly lower than that of the control group, indicating that downregulation of NDC80 protein significantly inhibited the invasive ability of glioblastoma cells.

## Discussion

Malignant brain tumors are among the most feared types of cancer because of their poor prognosis, as well as the direct repercussions on the quality of life and cognitive function (Omuro and DeAngelis, 2013). Malignant glioma is the most common type of primary malignant brain tumor, accounting for 80% of brain tumor patients and an annual incidence of 5.26 per 100,000 population or 17,000 new cases diagnosed per year (Dolecek et al., 2012). Glioblastoma, as a grade-Ⅳ glioma, is the most common and most malignant tumor in glioma. Because of the limited efficacy of conventional treatments, such as surgery, chemotherapy, and radiotherapy, there is an urgent need to identify a new and effective treatment. Over the past decade, many studies have made great progress elucidating the genetics and epigenetics of GBM (Polivka et al., 2017). Identification of relevant biomarkers for appropriate patient selection is essential for the successful development of novel therapies. In this study, we aimed to find potential biomarkers for predicting progression and prognosis in GBM.

In the present study, we obtained 716 DEGs common to three GMB gene expression datasets, which included 188 upregulated genes and 528 downregulated genes. These DEGs were associated with GO gene functions involving cellular protein modification, regulation of cell communication, and regulation of signaling. Many studies reported that glioma cells regulated cell communication and influenced signaling pathways (da Fonseca et al., 2016). The cells also formed networks, which allowed multicellular communication through microtube-associated gap junctions (Osswald et al., 2015). In addition, through KEGG pathway analysis, we found that these DEGs were involved in retrograde endocannabinoid and calcium signaling pathways and that the endocannabinoid pathway acted retrogradely and mediated synaptic modulation through the release of 2-arachidonoylglycerol, which also mediated long-term depression (Kano, 2014). Furthermore, many altered calcium-binding proteins have been observed in glioblastoma multiforme, which are implicated in the deregulation of calcium signaling and homeostasis in GBM (Polisetty et al., 2012). Glioblastoma can cause patients to have symptoms resembling epilepsy. Glioma cells have been shown to release pathological concentrations of glutamate, which are thought to play a role in tumor progression and the development of epilepsy (MacKenzie et al., 2016). Furthermore, the synaptic vesicle cycle involves important extracellular and endocytic processes, as well as protein complex formation and degradation processes, and has been reported to be associated with GBM (Gupta et al., 2014; Li and Kavalali, 2017).

We constructed a PPI network to investigate the interrelationship of the DEGs. Ten hub genes were identified, including *TOP2A*, *CDK1*, *BUB1*, *CDC20*, *BIRC5*, *MELK*, *KIF4A*, *PBK*, *NDC80*, and *TTK*. All of them were upregulated genes. Their gene expression was also validated *via* the TCGA and Oncomine databases. We conducted a survival analysis using patient data from the CGGA and the 10 hub genes. In our study, only six genes showed significant results: *TOP2A*, *CDK1*, *CDC20*, *BIRC5*, *MELK*, and *NDC80*. This finding implied that the prognosis of patients with glioblastoma could be predicted by detecting the expression level of these six genes. Furthermore, the results of this study provided potential biomarkers and target genes, which may be applied in the diagnosis and treatment of patients with glioblastoma for accurate therapy.

Interestingly, *TOP2A*, *CDK1*, *CDC20*, *MELK*, and *NDC80* genes were all associated with the cell cycle. The cell cycle involves multiple molecular pathways that appear to be the essential mechanism of the indefinite proliferation of malignant glioma. If the genes that regulate the cell cycle progression of malignant glioma are deregulated, the development of glioma will be promoted (Ouyang et al., 2016). Most of the genes identified here were reported as essential factors involved in cell division and proliferation. In mammals, *TOP2A* has an important role in altering DNA topology, and it is expressed in proliferating cells in the late S phase, with peak expression in the G2/M phases, which suggests that it has potential as a proliferation marker (Stiles and Rowitch, 2008). Compared with lower-grade astrocytomas and normal brain tissue, *TOP2A* transcription levels in GBM patients increased significantly, which is a useful prognostic indicator and may guide temozolomide chemotherapy (Arivazhagan et al., 2012). Cyclin-dependent kinase 1 (*CDK1*), located on 10q21.2, is one of the cyclin-dependent kinase genes that are important regulators of the cell cycle (Bertoni, 2018). *CDK1* has an important role in G1/S and G2/M phase transitions and promotes the M-phase process. In addition to glioma-related cell cycle regulators, there are many other cancer-related regulators, such as those found in lung adenocarcinoma and oral squamous cell carcinoma (Malumbres and Barbacid, 2009; Chen et al., 2015; Shi et al., 2016; Song et al., 2017). *CDC20*, a central regulator of the cell cycle in numerous cancers, was shown to have an essential role in the regulation of glioblastoma tumor-initiating cell proliferation, self-renewal, and survival (Xie et al., 2015a). *CDC20* knockdown by transduction with shCDC20 caused loss of tumor-initiating cells in the S, M, and G_2_ cell cycle phases and accumulation in the G_1_ phase (Xie et al., 2015b). In addition, silencing *CDC20* expression in tumor-initiating cells accelerated a significant increase in apoptotic cell death (Xie et al., 2015b). *BIRC5* (survivin) is associated with proliferation markers and histological malignancy grade, and its expression is inversely associated with prognosis (Varughese and Torp, 2016; Conde et al., 2017). Recent comprehensive studies have shown that knockdown of survivin in immortalized as well as in primary glioma cells leads to immense cellular polyploidy, with cells having a DNA content up to 32n, poly-merotelic kinetochore-microtubule connections, DNA damage, and the initiation of a DNA damage response (Hendruschk et al., 2011; Wiedemuth et al., 2014; Varughese et al., 2017). Knocking down *BIRC5* in GBM cells led to a transient G1 cell cycle arrest which was not able to halt the endoreplication of DNA (Conde et al., 2017). *MELK* is a member of a subfamily that activates serine/threonine protein kinases, and its expression increases with an increasing degree of malignancy in astrocytomas (Marie et al., 2008). Kig C showed that siRNA-mediated loss of *MELK* expression in glioblastoma cells caused a G1/S phase cell cycle arrest accompanied by cell death or a senescence-like phenotype, which implied that *MELK* inhibitors hold great potential for the treatment of glioblastomas alone or in combination with DNA-damaging therapies (Kig et al., 2017).

As one of the key elements of the outer kinetochore, NDC80, which has a molecular weight of 74 kDa, forms a heterotetrameric protein complex that plays an important role in cell mitosis (Suzuki et al., 2016). Abnormal expression of NDC80 protein causes chromosomal abnormalities, leading to instability of the genome, which is also a major factor in all tumorigenesis (Ju et al., 2017). Numerous studies have found that the components of the NDC80 complex are highly expressed in tumors, which can be used as a diagnostic marker for certain tumors and may even be an indicator for evaluating prognosis (Bieche et al., 2011). Therefore, the role of the NDC80 complex in the development of tumorigenesis has received increasing attention. However, *NDC80* and its proteins have rarely been reported in GBM. Our results showed that the expression of *NDC80* was upregulated in the GBM cell lines when compared to normal cells. Interestingly, the expression level of *NDC80* in glioma cells (U251, U-87MG, and A172) was significantly higher than that in normal astrocytes (HA 1800). Moreover, its expression level is positively correlated with the degree of malignancy. These results also suggest that *NDC80* plays a role in the pathogenesis and progression of GBM.

## Conclusion

In summary, the six genes identified here may be utilized to form a panel of disease progression and predictive biomarkers for GBM for clinical purposes. *NDC80*, one of the six genes, was discovered to have a potentially important role in GBM, a finding that needs to be further studied.

## Data Availability

The datasets presented in this study can be found in online repositories. The names of the repository/repositories and accession number(s) can be found in the article/Supplementary Material.

## Abbreviations

Glioblastoma (GBM), Gene Expression Omnibus (GEO), Gene Ontology (GO), Kyoto Encyclopedia of Genes and Genomes (KEGG), protein–protein interaction (PPI), differentially expressed genes (DEGs), The Cancer Genome Atlas (TCGA), World Health Organization (WHO), central nervous system (CNS), Gene Expression Profiling Interactive Analysis (GEPIA), the Chinese Glioma Genome Atlas (CGGA), reverse transcription-polymerase chain reaction (RT-PCR), and overall survival (OS).

## References

[B1] Arivazhagan A. Kumar D. M. Sagar V. Patric I. R. Sridevi S. Thota B. (2012). Higher topoisomerase 2 alpha gene transcript levels predict better prognosis in GBM patients receiving temozolomide chemotherapy: Identification of temozolomide as a TOP2A inhibitor. J. Neurooncol. 107 (2), 289–297. 10.1007/s11060-011-0758-3 22102081

[B2] Bertoni G. (2018). Cell cycle regulation by chlamydomonas cyclin-dependent protein kinases. Plant Cell 30 (2), 271. 10.1105/tpc.18.00103 29437987 PMC5868689

[B3] Bi J. Chowdhry S. Wu S. Zhang W. Masui K. Mischel P. S. (2020). Altered cellular metabolism in gliomas - an emerging landscape of actionable co-dependency targets. Nat. Rev. Cancer 20 (1), 57–70. 10.1038/s41568-019-0226-5 31806884

[B4] Bieche I. Vacher S. Lallemand F. Tozlu-Kara S. Bennani H. Beuzelin M. (2011). Expression analysis of mitotic spindle checkpoint genes in breast carcinoma: Role of NDC80/HEC1 in early breast tumorigenicity, and a two-gene signature for aneuploidy. Mol. Cancer 10, 23. 10.1186/1476-4598-10-23 21352579 PMC3058099

[B5] Chen X. Zhang F. H. Chen Q. E. Wang Y. Y. Wang Y. L. He J. C. (2015). The clinical significance of CDK1 expression in oral squamous cell carcinoma. Med. Oral Patol. Oral Cir. Bucal 20 (1), e7–12. 10.4317/medoral.19841 25129248 PMC4320424

[B6] Cheng W. Ren X. Zhang C. Cai J. Liu Y. Han S. (2016). Bioinformatic profiling identifies an immune-related risk signature for glioblastoma. Neurology 86 (24), 2226–2234. 10.1212/WNL.0000000000002770 27225222

[B7] Cloughesy T. F. Cavenee W. K. Mischel P. S. (2014). Glioblastoma: From molecular pathology to targeted treatment. Annu. Rev. Pathol. 9, 1–25. 10.1146/annurev-pathol-011110-130324 23937436

[B8] Conde M. Michen S. Wiedemuth R. Klink B. Schrock E. Schackert G. (2017). Chromosomal instability induced by increased BIRC5/Survivin levels affects tumorigenicity of glioma cells. BMC Cancer 17 (1), 889. 10.1186/s12885-017-3932-y 29282022 PMC5745881

[B9] da Fonseca A. C. C. Amaral R. Garcia C. Geraldo L. H. Matias D. Lima F. R. S. (2016). Microglia in cancer: For good or for bad? Adv. Exp. Med. Biol. 949, 245–261. 10.1007/978-3-319-40764-7_12 27714693

[B10] Das S. Meher P. K. Rai A. Bhar L. M. Mandal B. N. (2017). Statistical approaches for gene selection, hub gene identification and module interaction in gene Co-expression network analysis: An application to aluminum stress in soybean (Glycine max L.). PLoS One 12 (1), e0169605. 10.1371/journal.pone.0169605 28056073 PMC5215982

[B11] De Preter K. Vermeulen J. Brors B. Delattre O. Eggert A. Fischer M. (2010). Accurate outcome prediction in neuroblastoma across independent data sets using a multigene signature. Clin. Cancer Res. 16 (5), 1532–1541. 10.1158/1078-0432.CCR-09-2607 20179214

[B12] Dolecek T. A. Propp J. M. Stroup N. E. Kruchko C. (2012). CBTRUS statistical report: Primary brain and central nervous system tumors diagnosed in the United States in 2005-2009. Neuro. Oncol. 14 (5), v1–49. 10.1093/neuonc/nos218 23095881 PMC3480240

[B13] Giordano T. J. Kuick R. Else T. Gauger P. G. Vinco M. Bauersfeld J. (2009). Molecular classification and prognostication of adrenocortical tumors by transcriptome profiling. Clin. Cancer Res. 15 (2), 668–676. 10.1158/1078-0432.CCR-08-1067 19147773 PMC2629378

[B14] Gupta M. K. Jayaram S. Madugundu A. K. Chavan S. Advani J. Pandey A. (2014). Chromosome-centric human proteome project: Deciphering proteins associated with glioma and neurodegenerative disorders on chromosome 12. J. Proteome Res. 13 (7), 3178–3190. 10.1021/pr500023p 24804578

[B15] He W. Q. Gu J. W. Li C. Y. Kuang Y. Q. Kong B. Cheng L. (2015). The PPI network and clusters analysis in glioblastoma. Eur. Rev. Med. Pharmacol. Sci. 19 (24), 4784–4790. 26744869

[B16] Hendruschk S. Wiedemuth R. Aigner A. Topfer K. Cartellieri M. Martin D. (2011). RNA interference targeting survivin exerts antitumoral effects *in vitro* and in established glioma xenografts *in vivo* . Neuro. Oncol. 13 (10), 1074–1089. 10.1093/neuonc/nor098 21788344 PMC3177660

[B17] Huang D. W. Sherman B. T. Lempicki R. A. (2009). Systematic and integrative analysis of large gene lists using DAVID bioinformatics resources. Nat. Protoc. 4 (1), 44–57. 10.1038/nprot.2008.211 19131956

[B18] Ju L. L. Chen L. Li J. H. Wang Y. F. Lu R. J. Bian Z. L. (2017). Effect of NDC80 in human hepatocellular carcinoma. World J. Gastroenterol. 23 (20), 3675–3683. 10.3748/wjg.v23.i20.3675 28611520 PMC5449424

[B19] Kano M. (2014). Control of synaptic function by endocannabinoid-mediated retrograde signaling. Proc. Jpn. Acad. Ser. B Phys. Biol. Sci. 90 (7), 235–250. 10.2183/pjab.90.235 PMC4237895 25169670

[B20] Kig C. Beullens M. Beke L. Van Eynde A. Linders J. T. Brehmer D. (2017). Maternal embryonic leucine zipper kinase (MELK) reduces replication stress in glioblastoma cells. J. Biol. Chem. 292 (31), 12786. 10.1074/jbc.A113.471433 28778883 PMC5546021

[B21] Li Y. C. Kavalali E. T. (2017). Synaptic vesicle-recycling machinery components as potential therapeutic targets. Pharmacol. Rev. 69 (2), 141–160. 10.1124/pr.116.013342 28265000 PMC5394918

[B22] MacKenzie G. O'Toole K. K. Moss S. J. Maguire J. (2016). Compromised GABAergic inhibition contributes to tumor-associated epilepsy. Epilepsy Res. 126, 185–196. 10.1016/j.eplepsyres.2016.07.010 27513374 PMC5308901

[B23] Malumbres M. Barbacid M. (2009). Cell cycle, CDKs and cancer: A changing paradigm. Nat. Rev. Cancer 9 (3), 153–166. 10.1038/nrc2602 19238148

[B24] Marie S. K. Okamoto O. K. Uno M. Hasegawa A. P. Oba-Shinjo S. M. Cohen T. (2008). Maternal embryonic leucine zipper kinase transcript abundance correlates with malignancy grade in human astrocytomas. Int. J. Cancer 122 (4), 807–815. 10.1002/ijc.23189 17960622

[B25] Omuro A. DeAngelis L. M. (2013). Glioblastoma and other malignant gliomas: A clinical review. JAMA 310 (17), 1842–1850. 10.1001/jama.2013.280319 24193082

[B26] Osswald M. Jung E. Sahm F. Solecki G. Venkataramani V. Blaes J. (2015). Brain tumour cells interconnect to a functional and resistant network. Nature 528 (7580), 93–98. 10.1038/nature16071 26536111

[B27] Ouyang Q. Xu L. Cui H. Xu M. Yi L. (2016). MicroRNAs and cell cycle of malignant glioma. Int. J. Neurosci. 126 (1), 1–9. 10.3109/00207454.2015.1017881 26000816

[B28] Polisetty R. V. Gautam P. Sharma R. Harsha H. C. Nair S. C. Gupta M. K. (2012). LC-MS/MS analysis of differentially expressed glioblastoma membrane proteome reveals altered calcium signaling and other protein groups of regulatory functions. Mol. Cell. Proteomics 11 (6), M111.013565. 10.1074/mcp.M111.013565 PMC3433906 22219345

[B29] Polivka J. Jr. Polivka J. Holubec L. Kubikova T. Priban V. Hes O. (2017). Advances in experimental targeted therapy and immunotherapy for patients with glioblastoma multiforme. Anticancer Res. 37 (1), 21–33. 10.21873/anticanres.11285 28011470

[B30] Sabbagh Q. Andre-Gregoire G. Guevel L. Gavard J. (2020). Vesiclemia: Counting on extracellular vesicles for glioblastoma patients. Oncogene 39 (38), 6043–6052. 10.1038/s41388-020-01420-x 32801336

[B31] Saura M. Marquez S. Reventun P. Olea-Herrero N. Arenas M. I. Moreno-Gomez-Toledano R. (2014). Oral administration of bisphenol A induces high blood pressure through angiotensin II/CaMKII-dependent uncoupling of eNOS. FASEB J. 28 (11), 4719–4728. 10.1096/fj.14-252460 25103225

[B32] Shi Y. X. Zhu T. Zou T. Zhuo W. Chen Y. X. Huang M. S. (2016). Prognostic and predictive values of CDK1 and MAD2L1 in lung adenocarcinoma. Oncotarget 7 (51), 85235–85243. 10.18632/oncotarget.13252 27835911 PMC5356732

[B33] Song Z. Pan Y. Ling G. Wang S. Huang M. Jiang X. (2017). Escape of U251 glioma cells from temozolomide-induced senescence was modulated by CDK1/survivin signaling. Am. J. Transl. Res. 9 (5), 2163–2180. 28559969 PMC5446501

[B34] Stiles C. D. Rowitch D. H. (2008). Glioma stem cells: A midterm exam. Neuron 58 (6), 832–846. 10.1016/j.neuron.2008.05.031 18579075

[B35] Strepkos D. Markouli M. Klonou A. Piperi C. Papavassiliou A. G. (2020). Insights in the immunobiology of glioblastoma. J. Mol. Med. 98 (1), 1–10. 10.1007/s00109-019-01835-4 31650201

[B36] Stupp R. Dietrich P. Y. Kraljevic S. O. Pica A. Maillard I. Maeder P. Promising survival for patients with newly diagnosed glioblastoma multiforme treated with concomitant radiation plus temozolomide followed by adjuvant temozolomide. J. Clin. Oncol. (2002) 20(5):1375–1382. 10.1200/JCO.2002.20.5.1375 11870182

[B37] Suzuki A. Badger B. L. Haase J. Ohashi T. Erickson H. P. Salmon E. D. (2016). How the kinetochore couples microtubule force and centromere stretch to move chromosomes. Nat. Cell Biol. 18 (4), 382–392. 10.1038/ncb3323 26974660 PMC4814359

[B38] Szklarczyk D. Morris J. H. Cook H. Kuhn M. Wyder S. Simonovic M. (2017). The STRING database in 2017: Quality-controlled protein-protein association networks, made broadly accessible. Nucleic Acids Res. 45 (1), D362–D368. 10.1093/nar/gkw937 27924014 PMC5210637

[B39] Tan A. C. Ashley D. M. Lopez G. Y. Malinzak M. Friedman H. S. Khasraw M. (2020). Management of glioblastoma: State of the art and future directions. Ca. Cancer J. Clin. 70 (4), 299–312. 10.3322/caac.21613 32478924

[B40] Tang Z. Li C. Kang B. Gao G. Li C. Zhang Z. (2017). Gepia: A web server for cancer and normal gene expression profiling and interactive analyses. Nucleic Acids Res. 45 (1), W98–W102. 10.1093/nar/gkx247 28407145 PMC5570223

[B41] Varughese R. K. Skjulsvik A. J. Torp S. H. (2017). Prognostic value of survivin and DNA topoisomerase IIα in diffuse and anaplastic astrocytomas. Pathol. Res. Pract. 213 (4), 339–347. 10.1016/j.prp.2017.01.013 28214203

[B42] Varughese R. K. Torp S. H. (2016). Survivin and gliomas: A literature review. Oncol. Lett. 12 (3), 1679–1686. 10.3892/ol.2016.4867 27588117 PMC4998142

[B43] Ward A. Balwierz A. Zhang J. D. Kublbeck M. Pawitan Y. Hielscher T. (2013). Re-expression of microRNA-375 reverses both tamoxifen resistance and accompanying EMT-like properties in breast cancer. Oncogene 32 (9), 1173–1182. 10.1038/onc.2012.128 22508479

[B44] Wiedemuth R. Klink B. Topfer K. Schrock E. Schackert G. Tatsuka M. (2014). Survivin safeguards chromosome numbers and protects from aneuploidy independently from p53. Mol. Cancer 13, 107. 10.1186/1476-4598-13-107 24886358 PMC4041913

[B45] Xie Q. Wu Q. L. Mack S. C. Yang K. L. Kim L. Hubert C. G. CDC20 maintains tumor initiating cells. Oncotarget (2015) 6(15):13241–13254. 10.18632/oncotarget.3676 25938542 PMC4537011

[B46] Xie Q. Wu Q. Mack S. C. Yang K. Kim L. Hubert C. G. (2015). CDC20 maintains tumor initiating cells. Oncotarget 6 (15), 13241–13254. 10.18632/oncotarget.3676 25938542 PMC4537011

