# Supplementary figures and images for: Identification of core genes as potential biomarkers for predicting progression and prognosis in glioblastoma
Source: Front Genet. 2022 Sep 27;13:928407. doi: 10.3389/fgene.2022.928407 (PMC9552700; doi:10.3389/fgene.2022.928407)

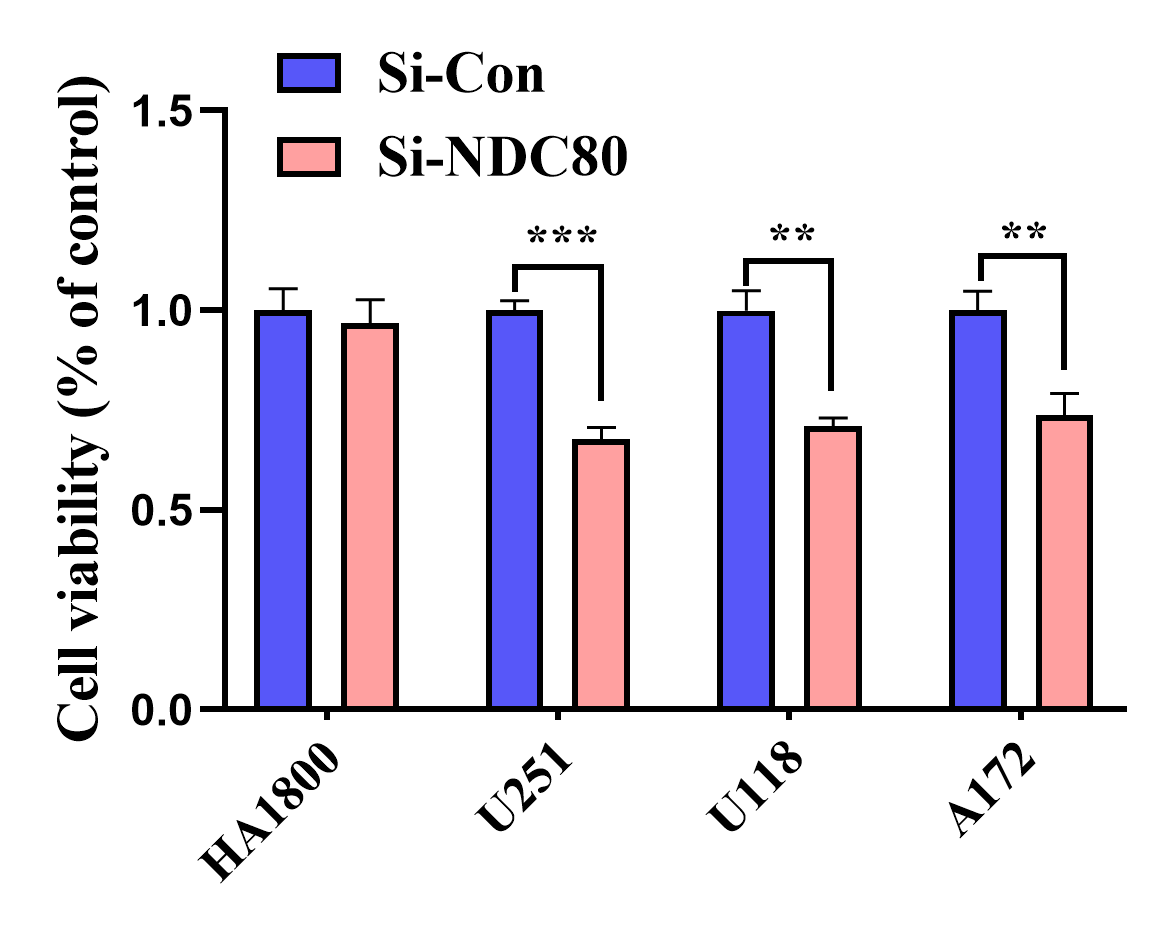

Supplement: Supplementary file 1 [file DataSheet1.ZIP › Raw data and figures/CCK8.tif]

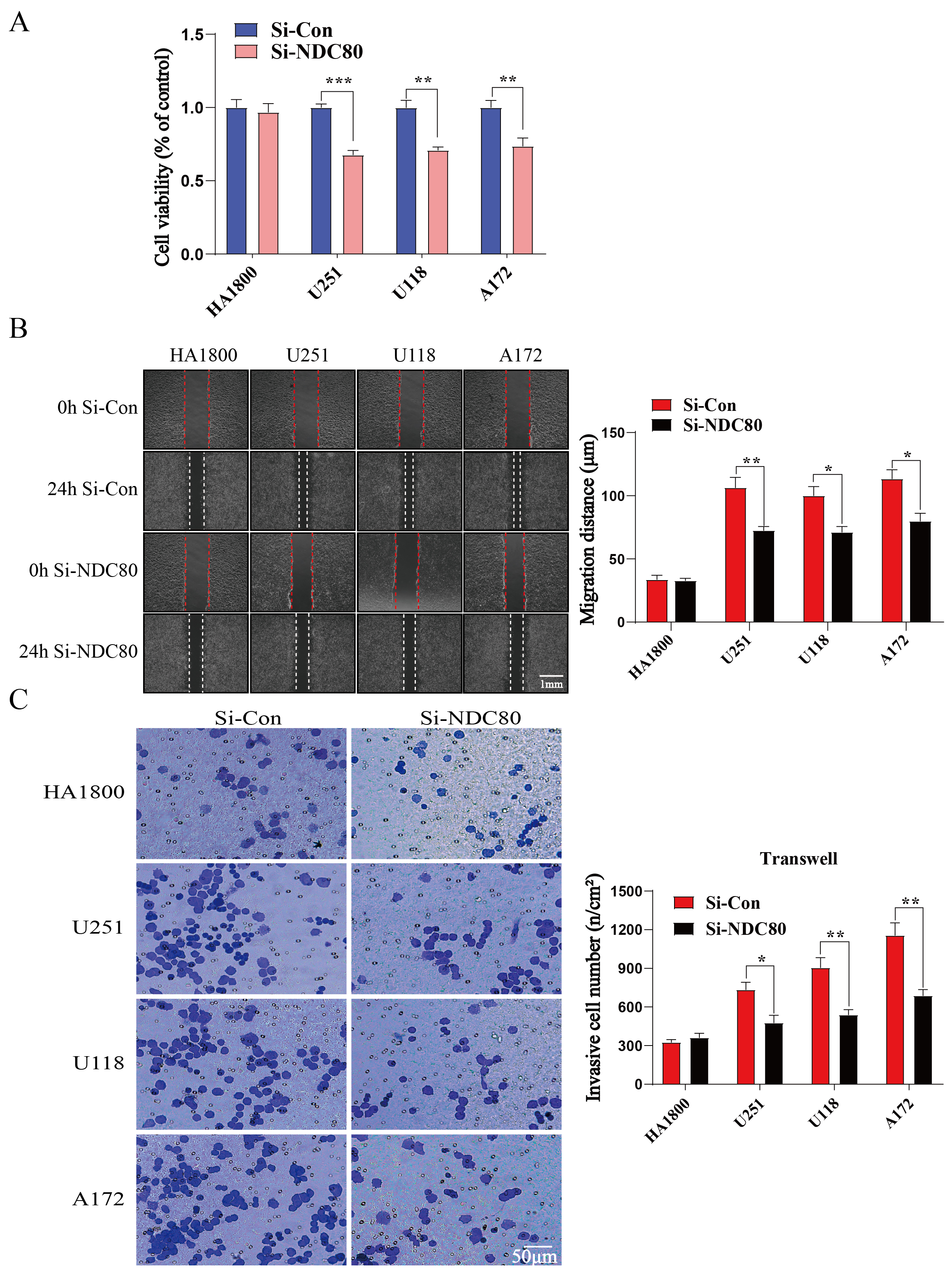

Supplement: Supplementary file 1 [file DataSheet1.ZIP › Raw data and figures/Figure 10.jpg]

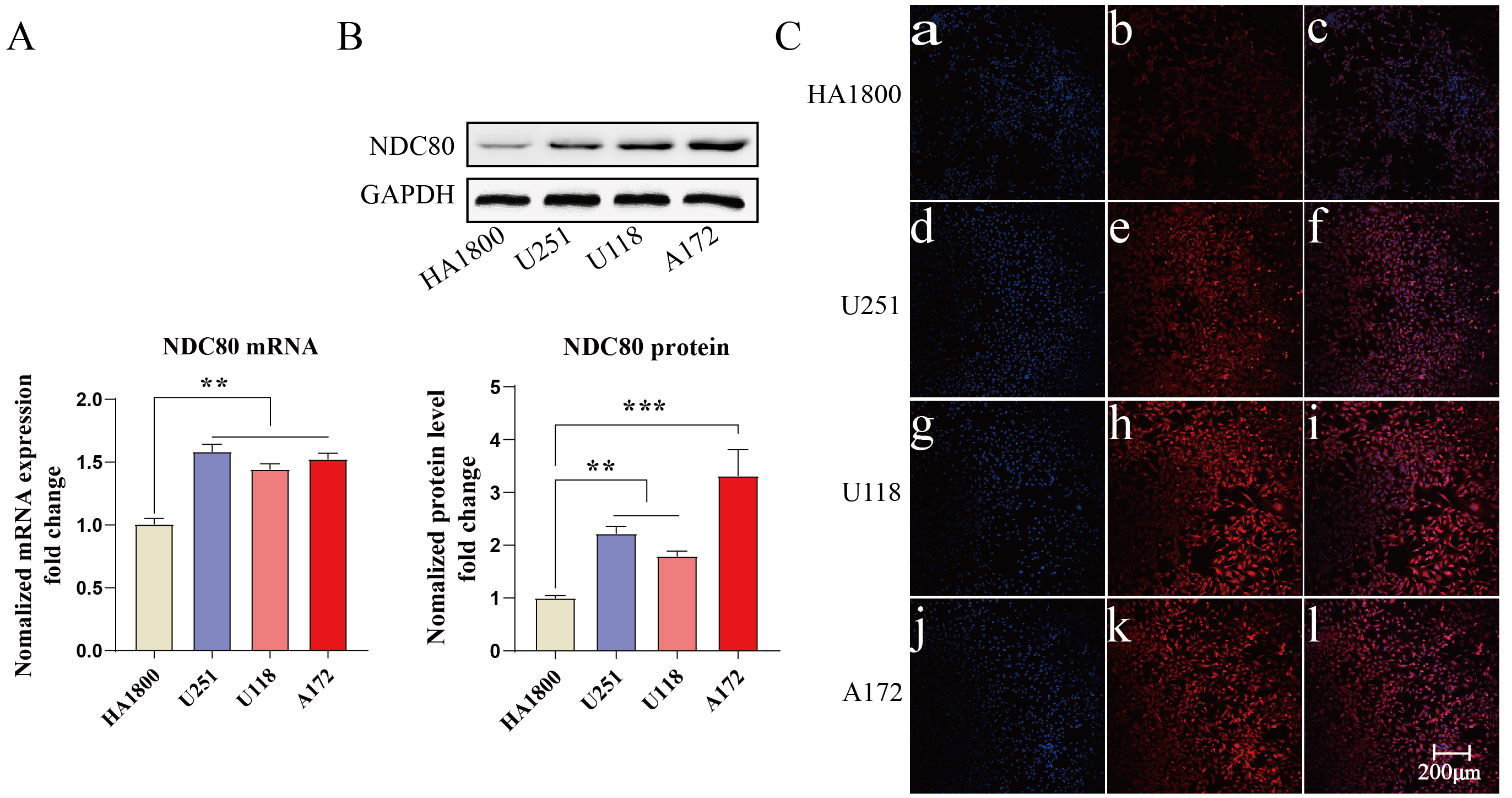

Supplement: Supplementary file 1 [file DataSheet1.ZIP › Raw data and figures/Figure 8.jpg]

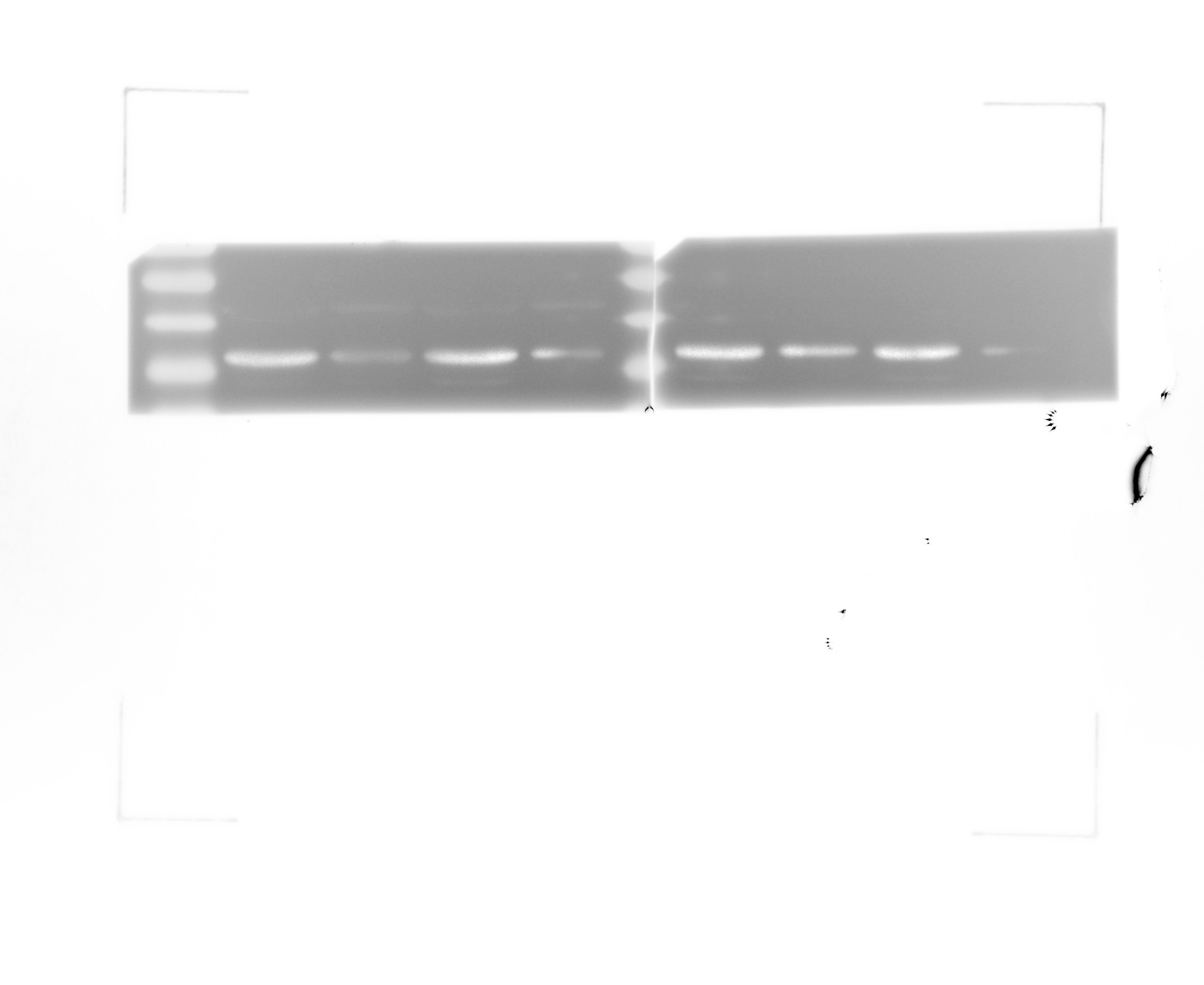

Supplement: Supplementary file 1 [file DataSheet1.ZIP › Raw data and figures/Figure 9 and raw data/fig9-NDC80 in gel location.jpg]

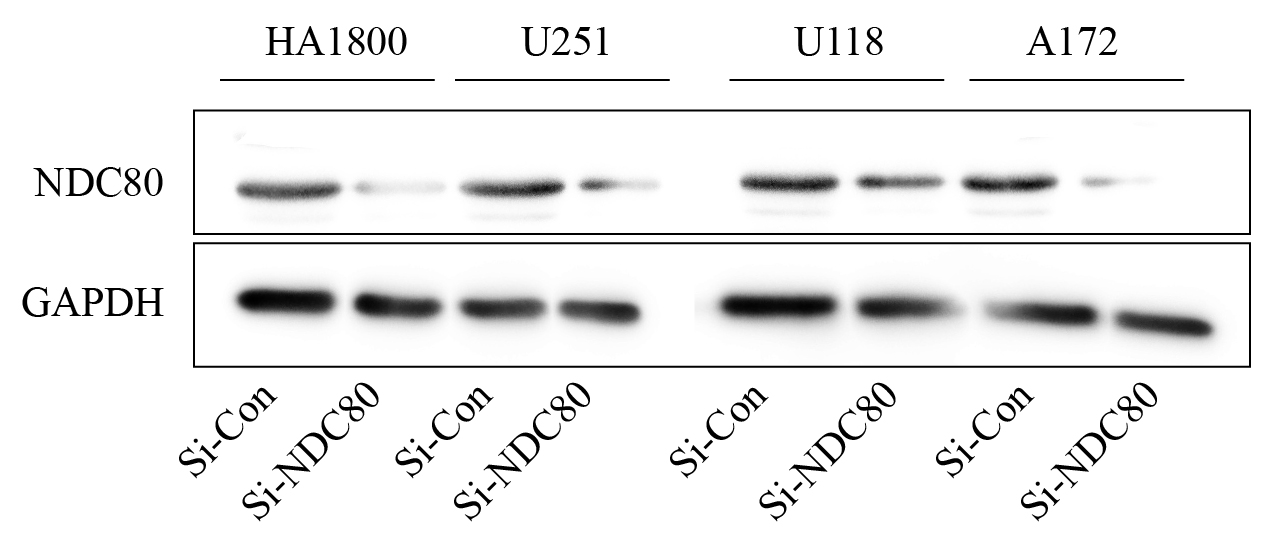

Supplement: Supplementary file 1 [file DataSheet1.ZIP › Raw data and figures/Figure 9 and raw data/Figure 9.jpg]

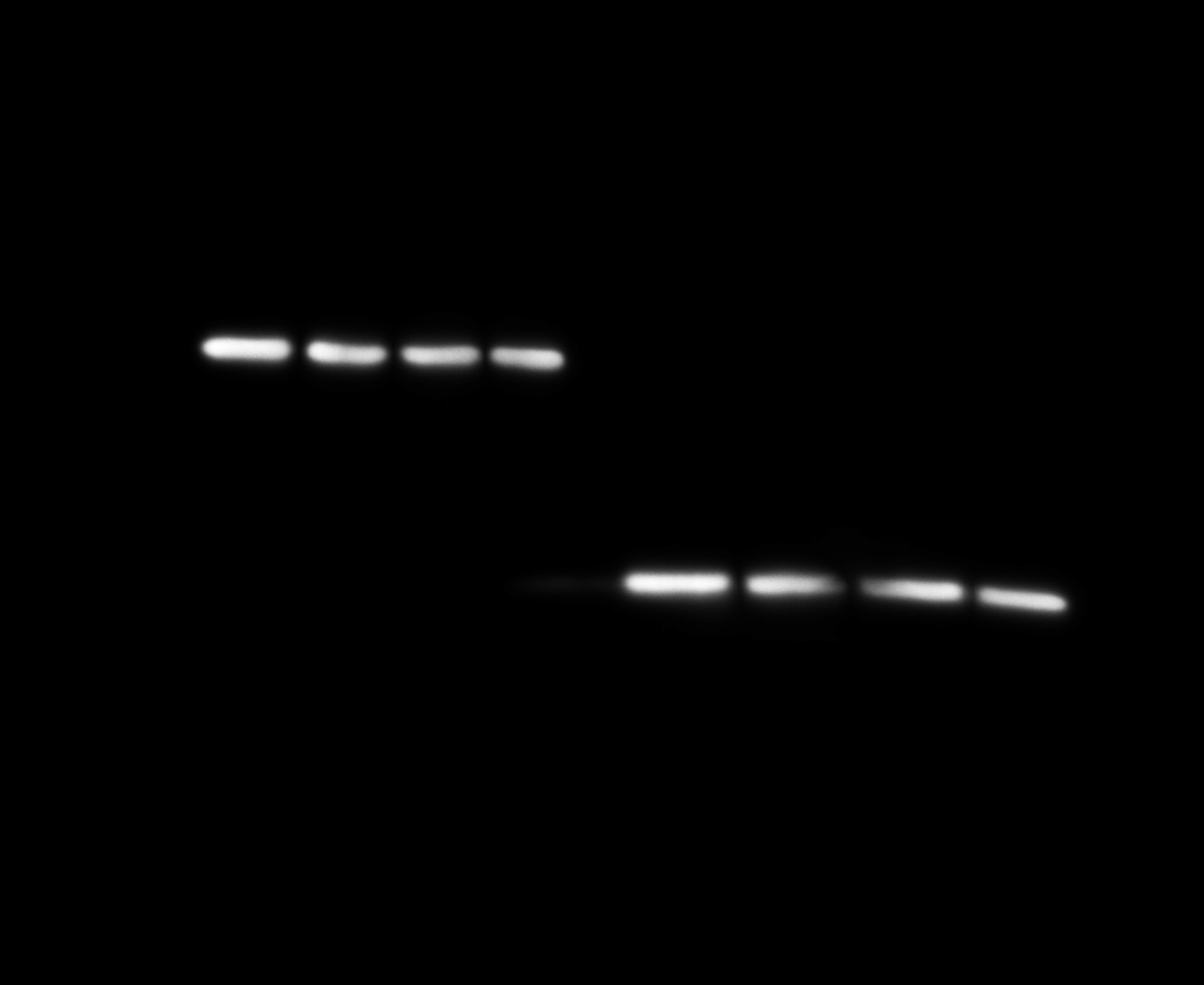

Supplement: Supplementary file 1 [file DataSheet1.ZIP › Raw data and figures/Figure 9 and raw data/GAPDH.jpg]

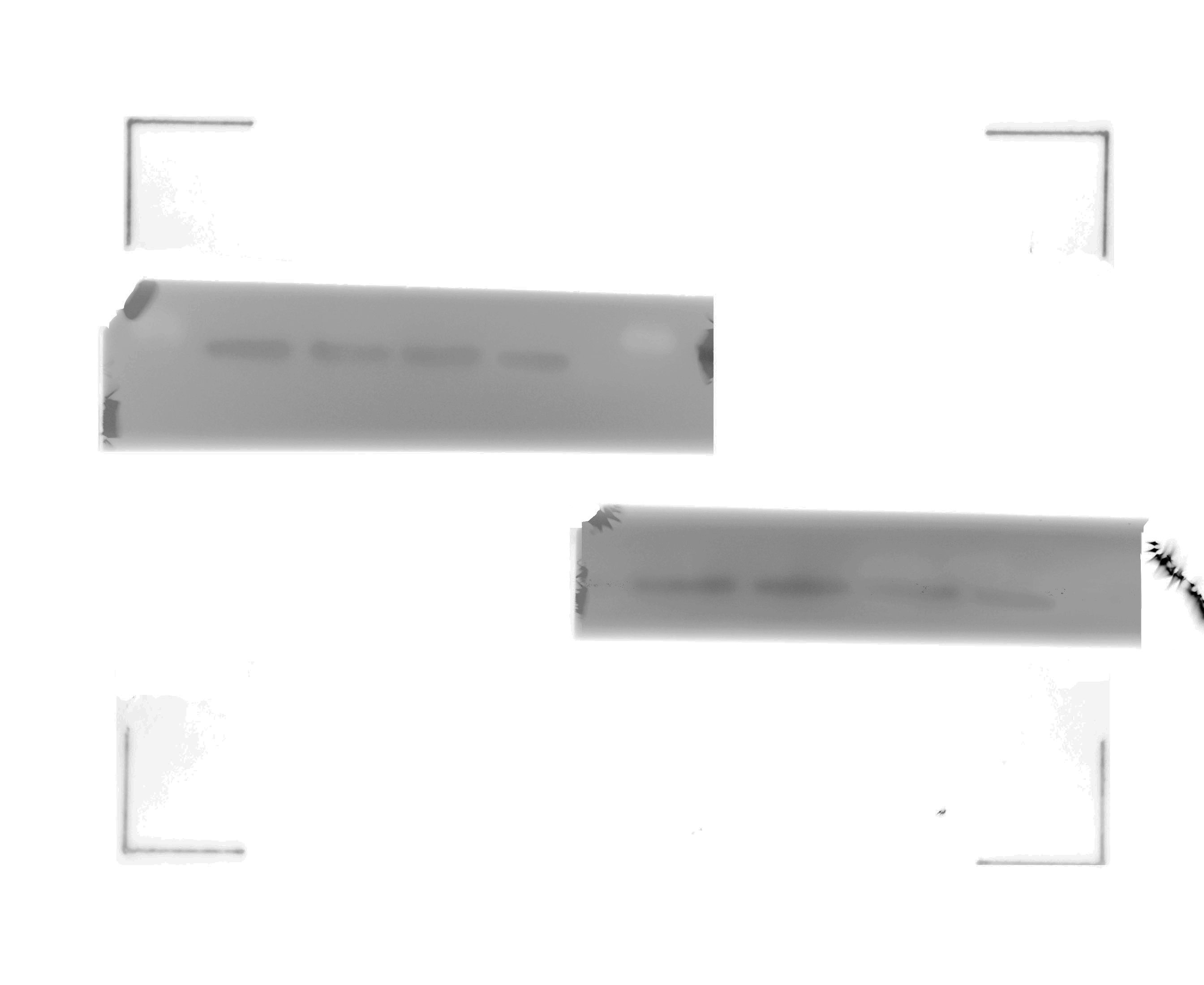

Supplement: Supplementary file 1 [file DataSheet1.ZIP › Raw data and figures/Figure 9 and raw data/gel-GAPDH.jpg]

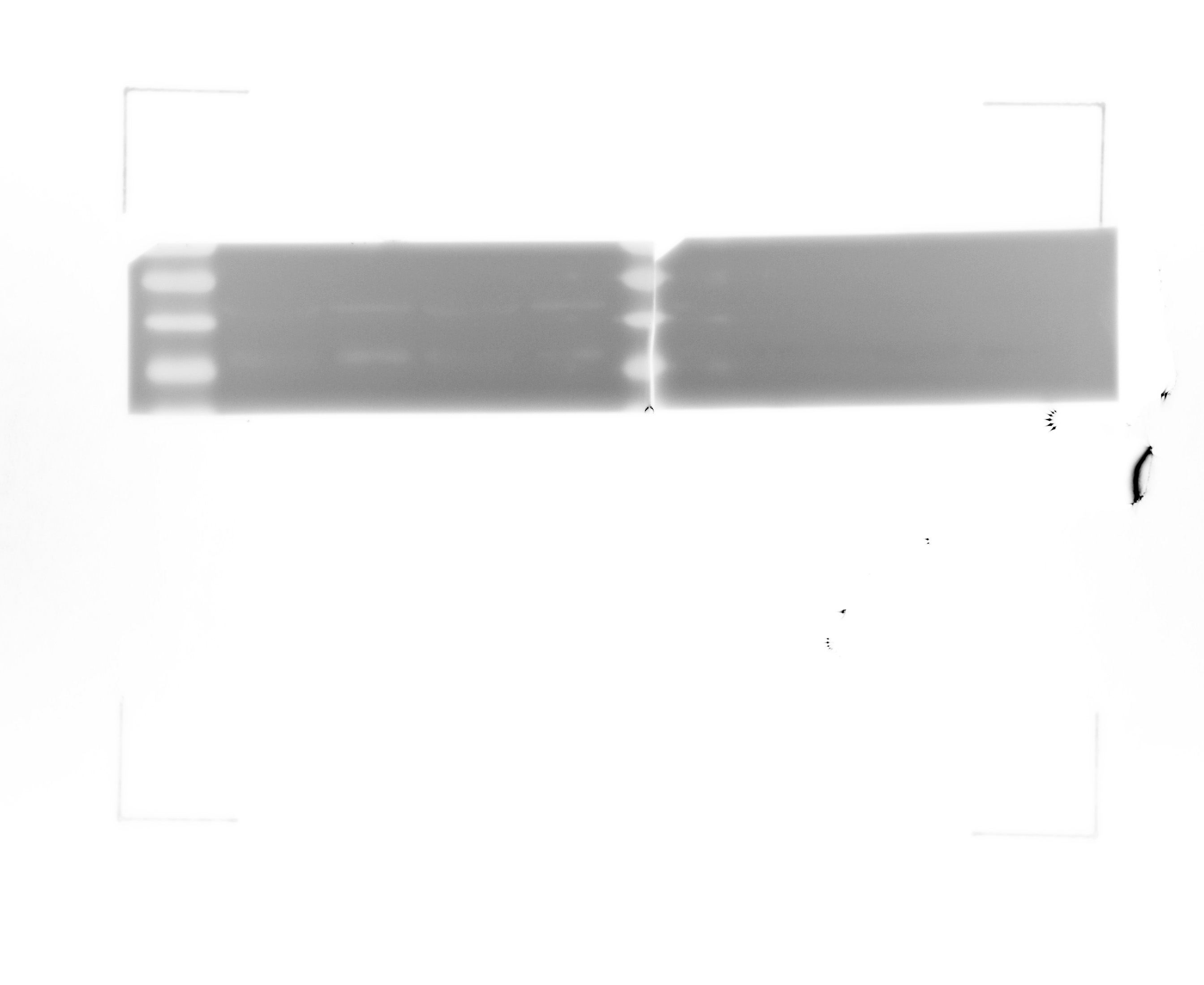

Supplement: Supplementary file 1 [file DataSheet1.ZIP › Raw data and figures/Figure 9 and raw data/gel-NDC80.jpg]

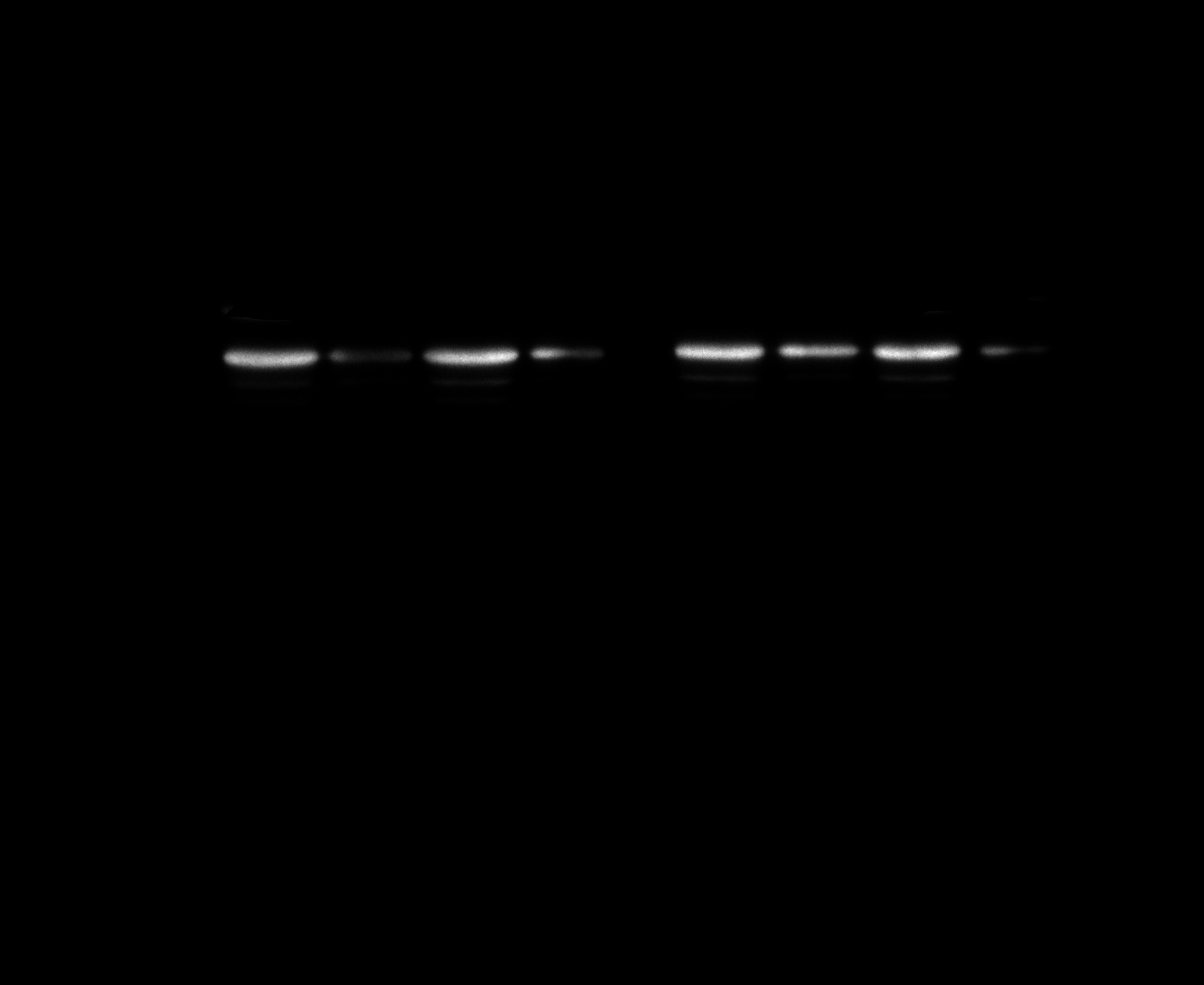

Supplement: Supplementary file 1 [file DataSheet1.ZIP › Raw data and figures/Figure 9 and raw data/NDC80.jpg]

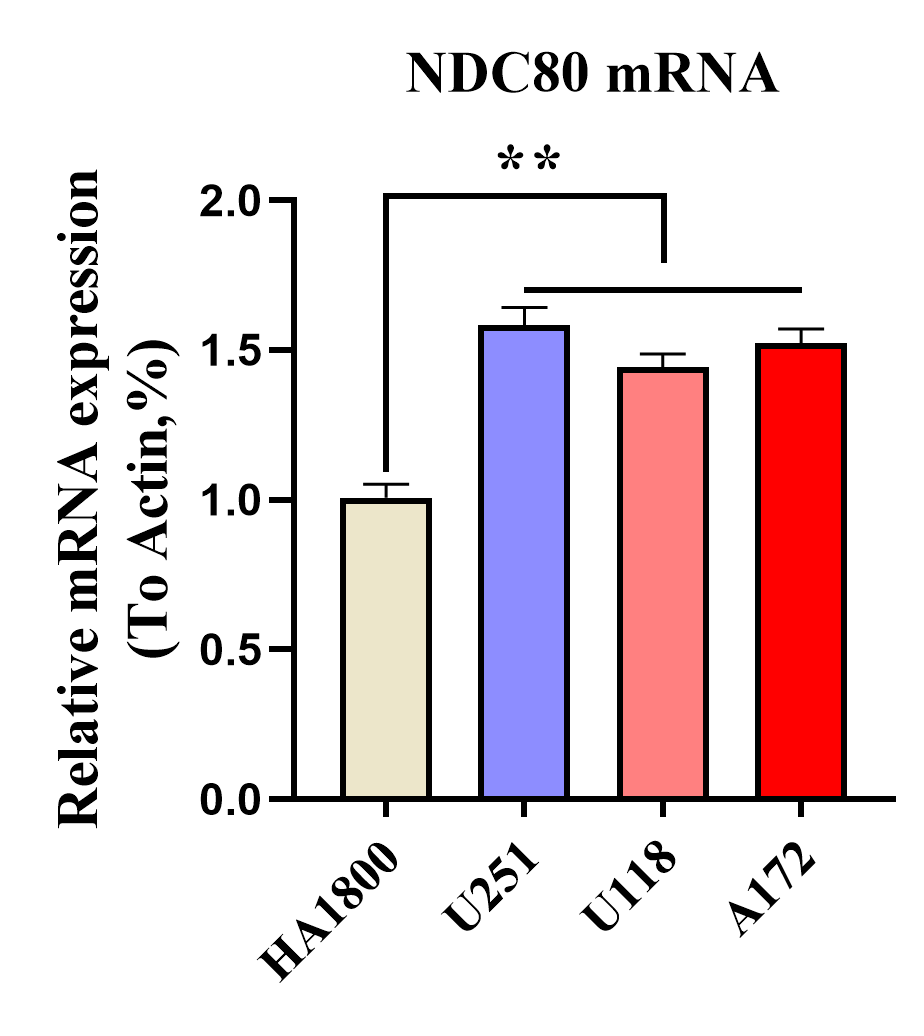

Supplement: Supplementary file 1 [file DataSheet1.ZIP › Raw data and figures/mRNA-expression.tif]

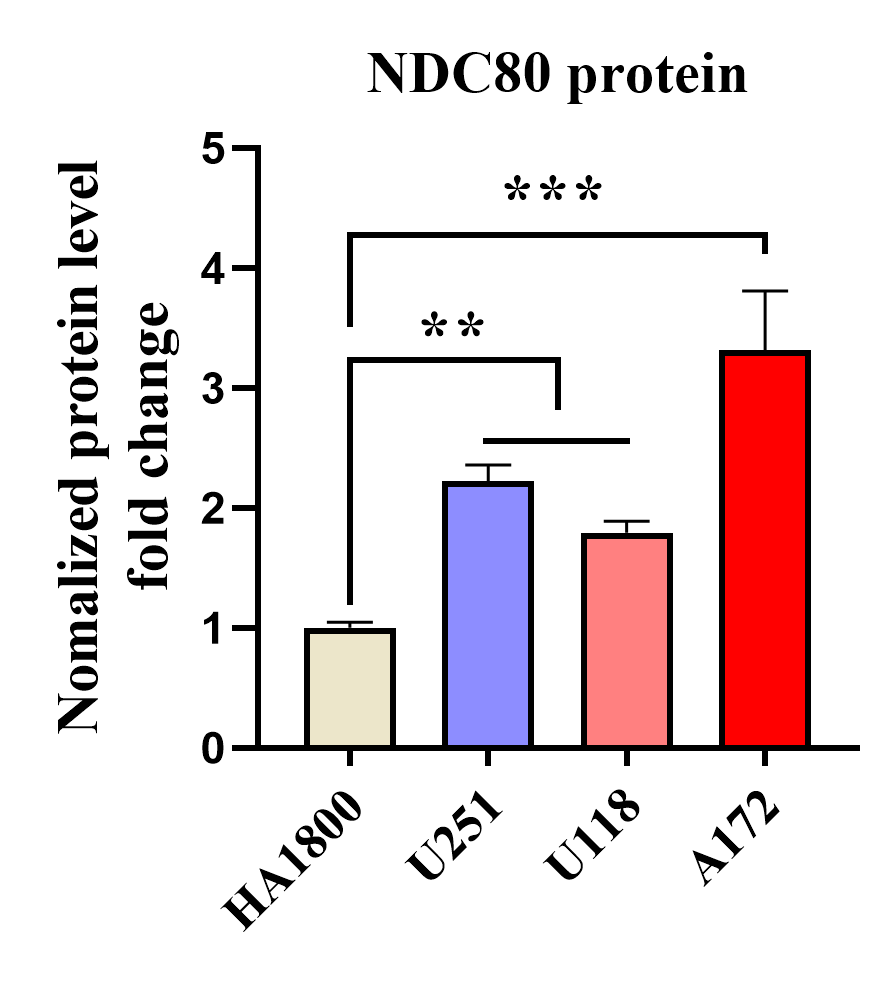

Supplement: Supplementary file 1 [file DataSheet1.ZIP › Raw data and figures/protein-expression.tif]

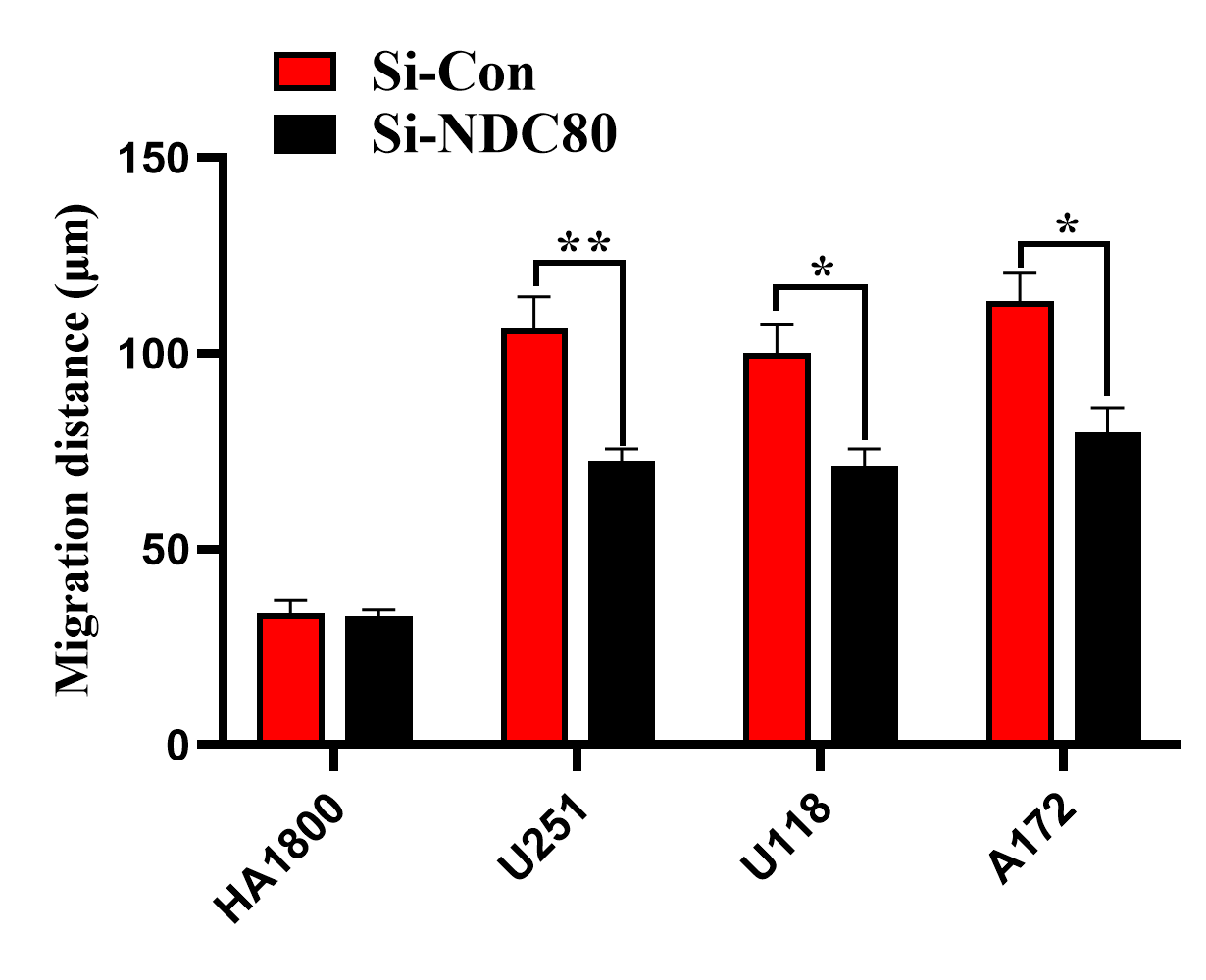

Supplement: Supplementary file 1 [file DataSheet1.ZIP › Raw data and figures/scratch-assay.tif]

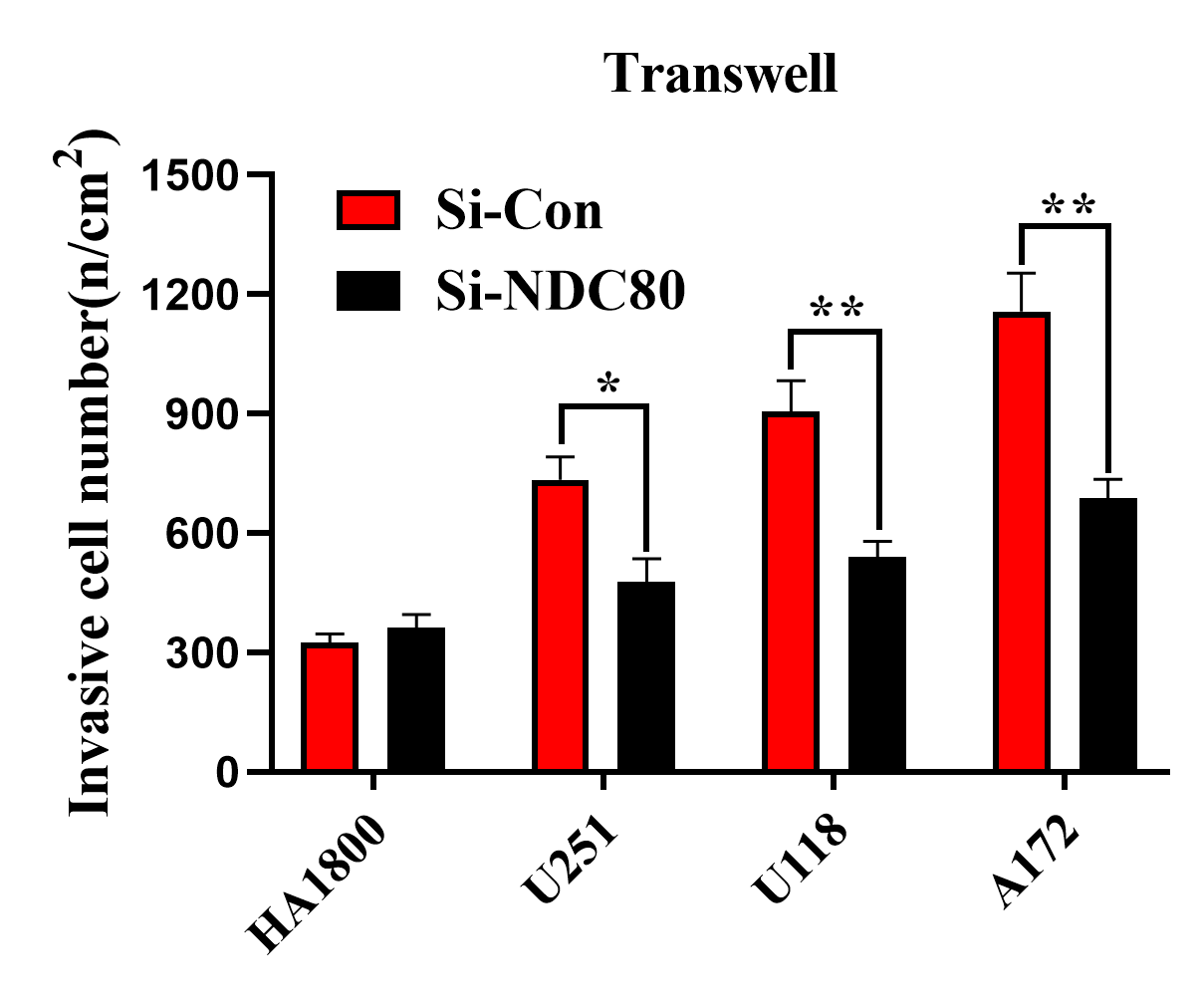

Supplement: Supplementary file 1 [file DataSheet1.ZIP › Raw data and figures/transwell.tif]

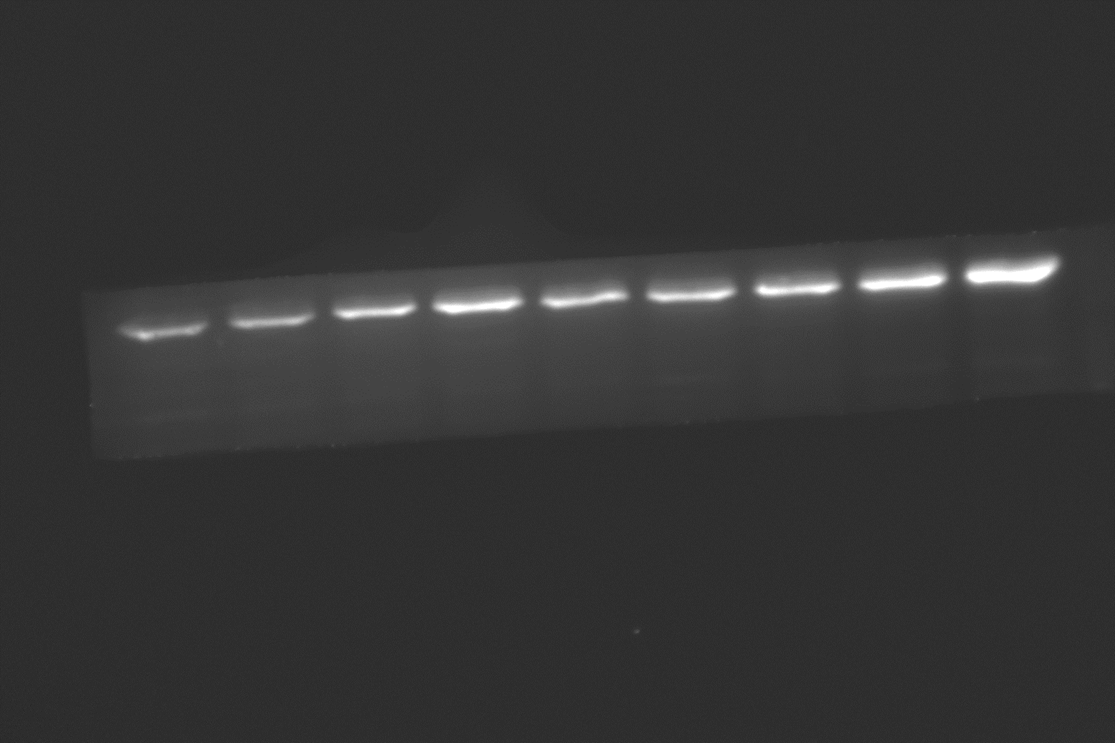

Supplement: Supplementary file 1 [file DataSheet1.ZIP › Raw data and figures/Uncropped western blot images/Raw-NDC80-expression.png]

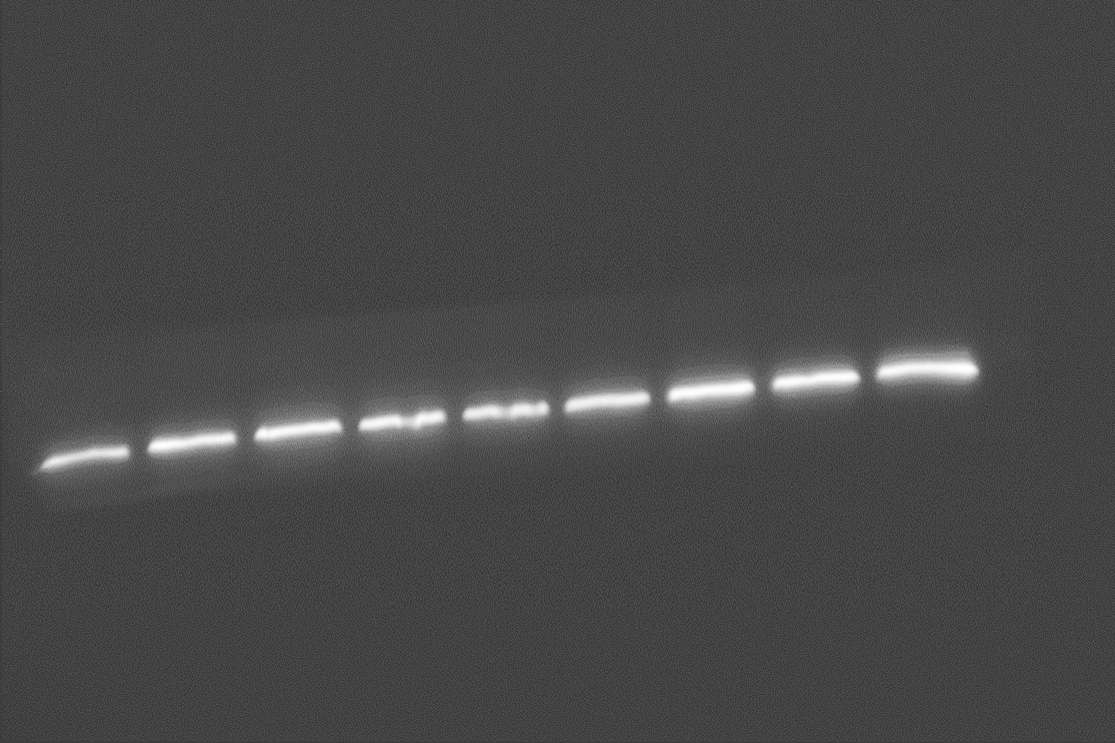

Supplement: Supplementary file 1 [file DataSheet1.ZIP › Raw data and figures/Uncropped western blot images/Raw-NDC80-GAPDH-expression.png]
